# Universal gut microbial relationships in the gut microbiome of wild baboons

**DOI:** 10.7554/eLife.83152

**Published:** 2023-05-09

**Authors:** Kimberly E Roche, Johannes R Bjork, Mauna R Dasari, Laura Grieneisen, David Jansen, Trevor J Gould, Laurence R Gesquiere, Luis B Barreiro, Susan C Alberts, Ran Blekhman, Jack A Gilbert, Jenny Tung, Sayan Mukherjee, Elizabeth A Archie

**Affiliations:** 1 Program in Computational Biology and Bioinformatics, Duke University Durham United States; 2 University of Groningen and University Medical Center Groningen, Department of Gastroenterology and Hepatology Groningen Netherlands; 3 University of Groningen and University Medical Center Groningen, Department of Genetics Groningen Netherlands; 4 Department of Biological Sciences, University of Notre Dame Notre Dame United States; 5 Department of Biology, University of British Columbia-Okanagan Campus Kelowna Canada; 6 Department of Ecology, Evolution, and Behavior, University of Minnesota Minneapolis United States; 7 Department of Biology, Duke University Durham United States; 8 Committee on Genetics, Genomics, and Systems Biology, University of Chicago Chicago United States; 9 Section of Genetic Medicine, Department of Medicine, University of Chicago Chicago United States; 10 Committee on Immunology, University of Chicago Chicago United States; 11 Department of Evolutionary Anthropology, Duke University Durham United States; 12 Duke University Population Research Institute, Duke University Durham United States; 13 Department of Pediatrics and the Scripps Institution of Oceanography, University of California, San Diego San Diego United States; 14 Department of Primate Behavior and Evolution, Max Planck Institute for Evolutionary Anthropology Leipzig Germany; 15 Departments of Statistical Science, Mathematics, Computer Science, and Bioinformatics & Biostatistics, Duke University Durham United States; 16 Center for Scalable Data Analytics and Artificial Intelligence, University of Leipzig Leipzig Germany; 17 Max Plank Institute for Mathematics in the Natural Sciences Leipzig Germany; Leibniz Institute on Aging Germany; Harvard T.H. Chan School of Public Health United States

**Keywords:** gut microbiota, microbiome community dynamics, correlations between bacteria, universality, personalization, longitudinal data analysis, *P. cynocephalus*

## Abstract

Ecological relationships between bacteria mediate the services that gut microbiomes provide to their hosts. Knowing the overall direction and strength of these relationships is essential to learn how ecology scales up to affect microbiome assembly, dynamics, and host health. However, whether bacterial relationships are generalizable across hosts or personalized to individual hosts is debated. Here, we apply a robust, multinomial logistic-normal modeling framework to extensive time series data (5534 samples from 56 baboon hosts over 13 years) to infer thousands of correlations in bacterial abundance in individual baboons and test the degree to which bacterial abundance correlations are ‘universal’. We also compare these patterns to two human data sets. We find that, most bacterial correlations are weak, negative, and universal across hosts, such that shared correlation patterns dominate over host-specific correlations by almost twofold. Further, taxon pairs that had inconsistent correlation signs (either positive or negative) in different hosts always had weak correlations within hosts. From the host perspective, host pairs with the most similar bacterial correlation patterns also had similar microbiome taxonomic compositions and tended to be genetic relatives. Compared to humans, universality in baboons was similar to that in human infants, and stronger than one data set from human adults. Bacterial families that showed universal correlations in human infants were often universal in baboons. Together, our work contributes new tools for analyzing the universality of bacterial associations across hosts, with implications for microbiome personalization, community assembly, and stability, and for designing microbiome interventions to improve host health.

## eLife digest

Communities of bacteria living in the guts of humans and other animals perform essential services for their hosts such as digesting food, degrading toxins, or fighting viruses and other bacteria that cause disease. These services emerge from so-called ‘ecological’ relationships between different species of bacteria.

One species, for example, may break down a molecule in human food into another compound that is, in turn, digested by another species into a small molecule that the human gut can absorb and use. The bacteria involved in such a process may become more or less common together in their host. In other situations, some bacteria may have opposing roles to each other, meaning that if one species becomes more abundant it may reduce the level of the other.

However, it is not known if relationships between different bacteria are consistent across hosts (i.e., universal) or unique to each host (personalized). In other words, if a pair of bacteria increase and decrease in abundance together in one host, do they do the same in other hosts? Microbes often swap genes with each other to gain new traits; as each host harbors a distinctive set of gut microbes, it may be possible for microbial relationships to change depending on the bacterial species present in a specific environment.

To investigate, Roche et al. studied the bacteria in thousands of samples of feces collected from 56 baboons over a 13-year period. These samples came from a long-term research project in Amboseli, Kenya which has been studying a population of wild baboons continuously since 1971.

Roche et al. measured the abundance of hundreds of gut bacteria in the feces to understand the relationships between pairs. This revealed that connections between species were largely universal rather than personalized to each baboon. Furthermore, the pairs of bacteria with the strongest positive or negative associations had the most consistent relationships across the baboons. Microbial relationships that have strong effects on the microbiome’s composition might therefore be especially universal.

Further analyses measuring gut bacteria in human babies also found that relationships between pairs of bacteria were largely universal. Hence, individual species of bacteria may fill similar ecological roles in each host across humans and other primates, and perhaps also in other mammals. These findings suggest that it may be possible to leverage the ecological relationships between bacteria to develop universal therapies for human diseases associated with gut bacteria, such as inflammatory bowel disease or *Clostridium difficile* infection.

## Introduction

Mammalian gut microbiomes are highly diverse, dynamic communities whose members exhibit the full spectrum of ecological relationships, from strong mutualisms like syntrophy and cross-feeding, to competition, parasitism, and predation (Faust and Raes, 2012; Foster and Bell, 2012; Dolinšek et al., 2016; Seth and Taga, 2014). These relationships mediate a variety of biological processes that have powerful effects on host health and fitness, including the metabolism of complex carbohydrates and toxins, and the synthesis of physiologically important compounds, like short-chain fatty acids, neurotransmitters, and vitamins (Faust and Raes, 2012; Foster and Bell, 2012; Dolinšek et al., 2016; Seth and Taga, 2014; Bäckhed et al., 2005; Gould et al., 2018; Pontrelli et al., 2022; Degnan et al., 2014). Despite their importance, major gaps remain in our understanding of microbial relationships in the gut (Faust and Raes, 2012; Loftus et al., 2021; Bashan et al., 2016). We typically do not know if the abundance of one microbe consistently predicts the abundance of other microbes in the same host community, nor do we understand whether these correlative relationships are consistent in strength or direction across hosts (Bashan et al., 2016; Widder et al., 2016; Cao et al., 2017; Faust and Raes, 2016).

Knowing the overall direction and strength of these correlative relationships is important to understanding the ecological relationships that mediate gut microbial processes and shape gut microbiome assembly, stability, and productivity (Coyte et al., 2015; Palmer and Foster, 2022; Hu et al., 2022). For instance, sets of microbes that exhibit strong, positive relationships within hosts may represent networks of cooperating taxa that promote each other’s growth (Bäckhed et al., 2005; Loftus et al., 2021; Wu et al., 2021). In turn, these strong, mutualistic interdependencies can create an ecological house of cards where microbes rise and fall together, hampering community assembly and stability (Coyte et al., 2015; Coyte et al., 2021). Further, understanding the degree to which correlative relationships between microbes are the same or different in different hosts can shed light on whether hosts share similar, underlying microbial ecologies (Loftus et al., 2021; Bashan et al., 2016; Gao et al., 2020; Vila et al., 2020; San-Juan-Vergara et al., 2018). Microbial ecologies that are similar across hosts may make it possible to manipulate the microbiome’s emergent properties to improve host health (Loftus et al., 2021; Bashan et al., 2016; Cao et al., 2017; Coyte et al., 2015; Coyte et al., 2021; Gonze et al., 2018).

To date, there are several reasons to think that correlative relationships in the gut microbiome will not be consistent across hosts and will instead be individualized to each host. For instance, several common community and evolutionary processes—such as horizontal gene transfer and priority effects—can lead microbiome taxa to fill different ecological roles in different hosts (Dolinšek et al., 2016; Franzosa et al., 2015; Faith et al., 2013; Bik et al., 2016; Caporaso et al., 2011; Costello et al., 2009). Further, genotype by environment interactions and plasticity could lead some microbes to adopt context-dependent metabolisms and ecological roles depending on their microbial neighbors or other aspects of the environment (Louca et al., 2018; Rainey and Quistad, 2020; Martiny et al., 2015; Debray et al., 2022). Finally, the common observation that gut microbial community compositions (i.e. the presence and abundance of taxa) are highly individualized is sometimes attributed to host-specific microbial ecologies and relationships (Franzosa et al., 2015; Faith et al., 2013; Bik et al., 2016; Caporaso et al., 2011; Costello et al., 2009; Risely et al., 2021; Kolodny et al., 2019; Flores et al., 2014; Johnson et al., 2019; Pruss et al., 2021).

**Figure 1.**
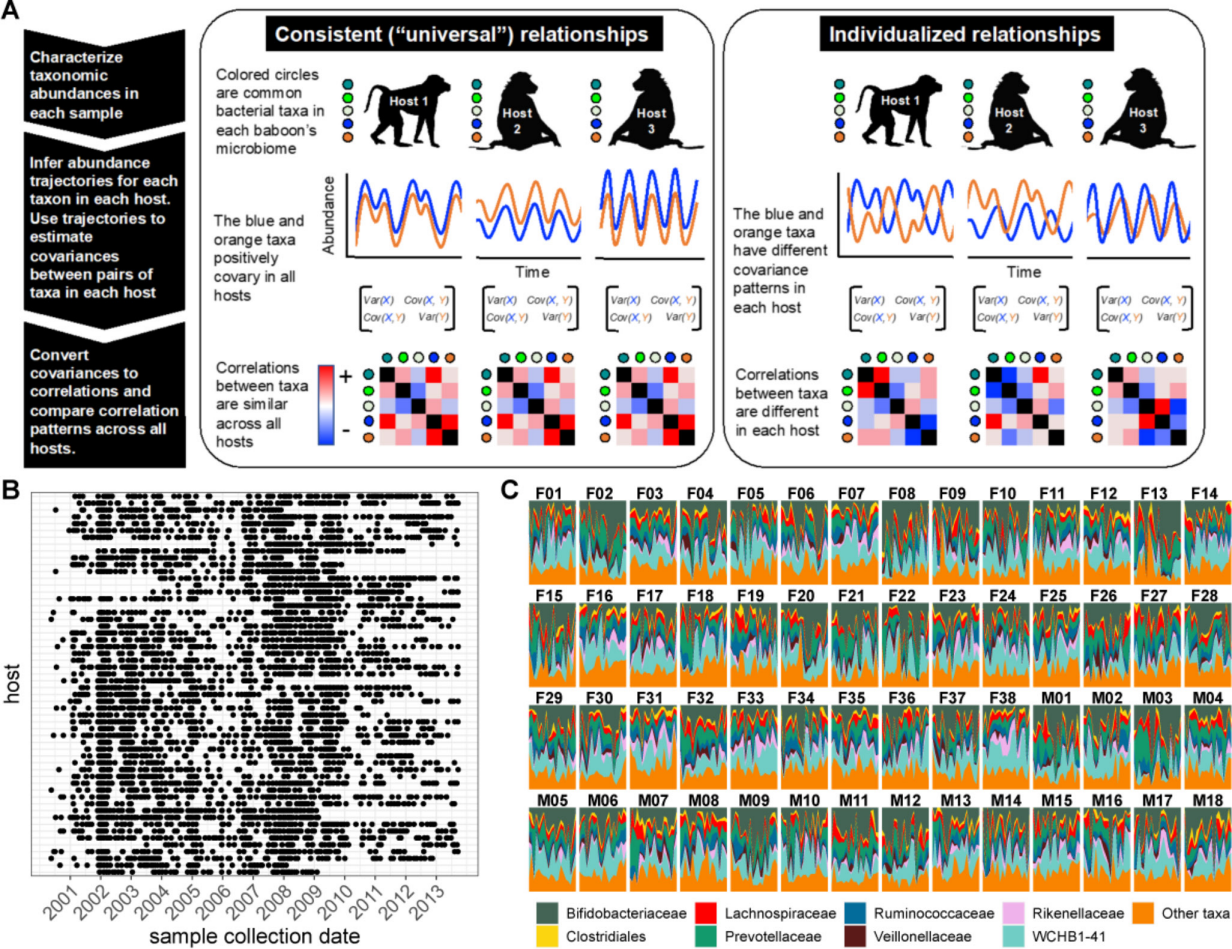
Testing the generalizability of gut microbial correlations across hosts. (**A**) Schematic illustrating our approach for testing the degree to which gut microbial abundance correlations are consistent (i.e. ‘universal’; Bashan et al., 2016) across different baboon hosts. The left-hand set of images show our expectations for consistent correlation patterns; the right-hand images show our expectation for individualized correlation patterns. Colored circles next to each baboon represent microbes found in at least 50% of samples overall (and at least 20% of samples within each host). We also excluded putative duplicate 16S gene copies (see Methods). In each host, we inferred centered log-ratio (CLR) abundance trajectories for these taxa using a multinomial logistic-normal modeling approach implemented in the R package ‘fido’ (Silverman et al., 2022). Cartoons of two such trajectories for the orange and blue taxa are below each baboon. We used these trajectories to infer covariances between each pair of taxa in all baboons (represented by covariance matrices; we only analyzed microbial pairs whose joint zero abundance was less than 5% of all samples across hosts). We then converted these covariances to Pearson’s correlations and compared bacterial correlation patterns across all hosts, shown as heat maps (red cells are positively correlated taxa; blue cells reflect negatively correlated taxa). (**B**) Irregular time series of fecal samples used to infer microbial CLR abundance trajectories in 56 baboon hosts (*n*=5534 total samples; 75–181 samples per baboon across 6–13.3 years). Each point represents a fecal sample collected from a known individual baboon (*y*-axis) on a given date (*x*-axis). Samples from the same baboon were collected a median of 20 days apart (range = 0–723 days; 25th percentile = 7 days, 75th percentile = 49 days). (**C**) Relative abundances of the eight most prevalent gut bacterial orders and families over time (x-axis) for all 56 hosts (samples from females are labeled with an F; male samples with an M). Microbiota composition was somewhat individualized to each host (Figure 1—figure supplement 2; Björk et al., 2022; Grieneisen et al., 2021).

However, to date, the handful of studies that have tested the generalizability of gut microbial relationships across hosts suggest that microbiome community ecology is not highly individualized and is instead largely consistent (i.e. ‘universal’) across hosts (Figure 1A; Bashan et al., 2016; Gao et al., 2020; Vila et al., 2020; San-Juan-Vergara et al., 2018; Kalyuzhny et al., 2017). For instance, Bashan et al., 2016, inferred ‘universal’ gut microbial relationships in the human gut microbiome by applying dissimilarity overlap analysis (DOA) to cross-sectional samples from several human data sets. DOA infers universal microbial relationships by testing whether pairs of hosts who share many of the same microbiome taxa also have similar abundances of those taxa (Bashan et al., 2016; Gao et al., 2020; Vila et al., 2020; San-Juan-Vergara et al., 2018; Kalyuzhny et al., 2017). This approach relies on the assumption that, when two communities share many of the same species and have similar abundances of those species, they do so because of a shared, underlying set of between-species bacterial abundance relationships (Bashan et al., 2016; Kalyuzhny et al., 2017). While many studies using this approach find evidence that microbial relationships are ‘universal’ (Bashan et al., 2016; Gao et al., 2020; Vila et al., 2020; San-Juan-Vergara et al., 2018), DOA’s assumptions have been questioned because environmental gradients, stochastic processes, and the presence of many non-interactive species can lead to the spurious detection of universality (Bashan et al., 2016; Kalyuzhny et al., 2017; Marsland et al., 2020).

**Figure 1—figure supplement 1.**
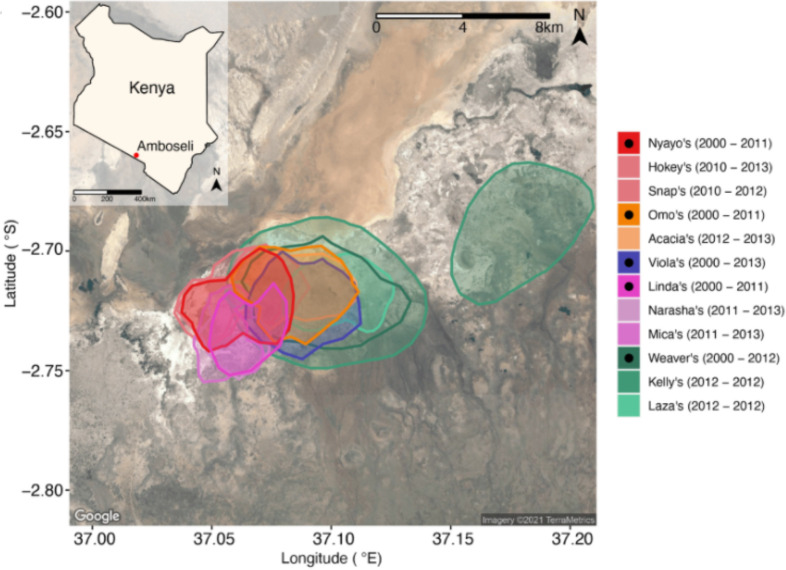
Host ranging patterns. Ranging patterns for the baboon social groups included in this study during the period of microbiome sampling (May 19, 2000, to September 19, 2013). See Björk et al., 2022, for an in-depth analysis of environmental drivers of microbiome dynamics in this host population.

**Figure 1—figure supplement 2.**
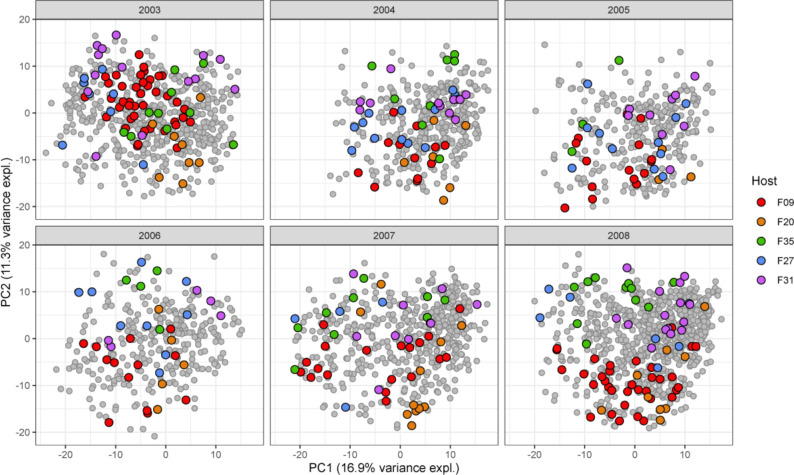
Variation across hosts. Each baboon exhibits a somewhat distinctive gut microbial community (p<0.0001; ANOVA) summarized here by the first two principal components of centered log-ratio (CLR)-transformed amplicon sequence variant (ASV) abundances of ASVs during six representative years of sample collection (see Figure 1B). Colored dots represent one of five especially well-sampled hosts, and colors indicate samples from the same host. Gray dots represent all other samples collected in the same year.

**Figure 1—figure supplement 3.**
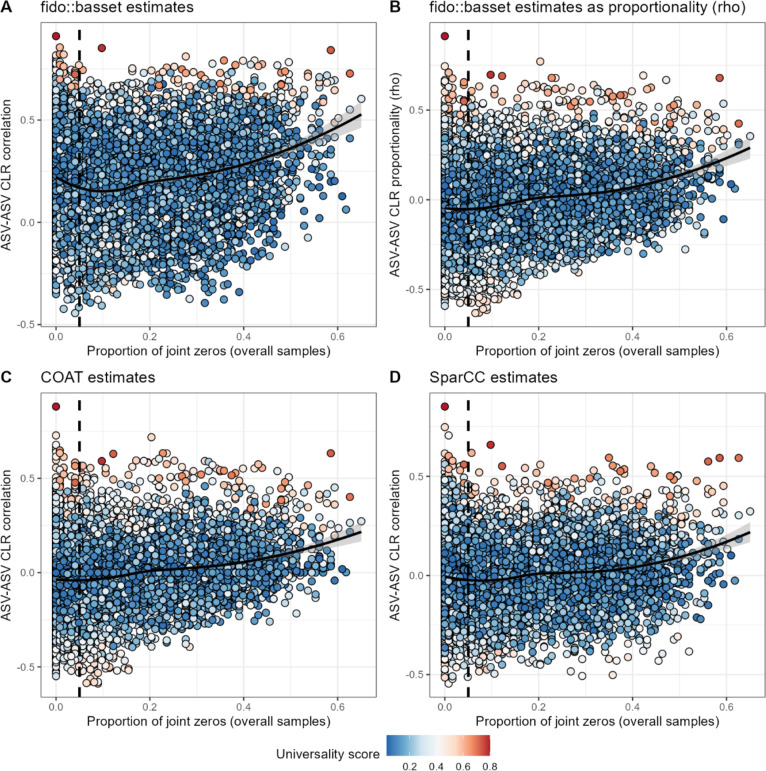
An increase in frequency of joint zeros is associated with positive ASV-ASV correlations across four methods. ASV-ASV pairs with a high frequency of joint absences (zero abundance observations) are more likely to be estimated as positively correlated by four methods: (**A**) *basset*, reporting Pearson’s correlation; (**B**) *basset*, reporting proportionality (*rho*; see Quinn et al., 2017); (**C**) COAT (see Cao et al., 2019); or (**D**) SparCC (see Friedman and Alm, 2012). The vertical dashed line in all panels shows a 0.05 cutoff for the proportion of joint zeros, the cutoff used in the main text. ASV, amplicon sequence variant.

An obvious alternative is to measure microbial correlations directly from microbiome time series from several hosts (Loftus et al., 2021; Faust et al., 2015). Unlike DOA, this approach should be able to pinpoint which microbiome taxa exhibit the most and least consistent relationships across hosts. However, measuring microbial correlations from longitudinal, multi-host microbiome time series has its own challenges: time series with adequately dense sampling are rare, and most such data sets exhibit temporal autocorrelation and irregular sampling (Faust et al., 2015). Further, the most common, and still most feasible, way to collect microbiome community data—via high-throughput sequencing—generates noisy count data that usually can only be interpreted in terms of relative (not absolute) abundances (Gloor et al., 2017; Quinn et al., 2017). Finally, correlation cannot be used to infer causality, and in the absence of experiments, we cannot differentiate whether microbial correlation patterns arise from ecological interactions (e.g. competition, predation, facilitation) or shared responses to the environment.

To address several of these challenges, here we combine extensive time series data on the stool-associated microbiota with a multinomial logistic-normal modeling framework (Figure 1; *n*=5534 samples from 56 baboons; 75–181 samples per baboon across 6–13.3 years, between 2000 and 2013; Alberts and Altmann, 2012; Björk et al., 2022; Grieneisen et al., 2021). This framework uses 16S rRNA sequencing count data to learn a smoothly evolving Gaussian process. The baboons were the subject of long-term research on individually recognized animals by the Amboseli Baboon Research Project in Kenya, which has been studying baboon ecology and behavior in the Amboseli ecosystem since 1971 (Alberts and Altmann, 2012). The baboons range over the same habitat and experience similar diets and sources of microbial colonization, facilitating inference about the consistency of microbial correlations across hosts (Figure 1—figure supplement 1; Björk et al., 2022; Grieneisen et al., 2021). To partly account for environmental drivers of microbial dynamics, our modeling approach controls for variation attributable to seasonal changes in the animals’ diets, proportionality in the count data, and irregularity in sampling to produce per-individual, per-taxon trajectories of log-ratio abundances that we used to estimate pairwise microbial correlations within each host.

We pursued five main objectives. First, we characterized the overall sign and strength of pairwise correlations in bacterial abundance within each host. Second, we tested the degree to which these correlation patterns are systematically consistent across hosts or individualized by host (Figure 1A). Third, we identified phylogenetic and host-related predictors of the direction and universality of bacterial correlations, including phylogenetic relationships between microbes, host age, and host genetic relatedness. Fourth, we tested whether the microbial correlations we observed were driven by shared responses to host diets or seasonality, or by synchronized microbial dynamics across hosts. Fifth, we tested the generalizability of our findings by comparing the patterns of universality in our data set to two microbiome time series from humans (Johnson et al., 2019; Vatanen et al., 2016).

Our predictions for these analyses were influenced by ideas from community and microbial ecology. First, because strong interdependencies can hamper community assembly and destabilize community dynamics (Coyte et al., 2015; Palmer and Foster, 2022; Hu et al., 2022; Coyte et al., 2021), we expected that most microbial correlations would be weak with few strong positive relationships. Second, consistent with studies that used DOA, we expected that microbial relationships would be more consistent across hosts than individualized (see Figure 1A for a visualization of this prediction). This result would suggest that personalized microbiota—their compositions and dynamics—do not arise from host-specific microbiome ecologies (Bashan et al., 2016; Gao et al., 2020; Vila et al., 2020; San-Juan-Vergara et al., 2018). Third, we expected to observe positive correlations between taxa that are close phylogenetic relatives. This is because related bacteria may have similar functional properties and hence similar ecological relationships with other members of the community. They may also have dynamics that are driven by similar selective forces imposed by the host or host’s environment. Alternatively, competitive exclusion may lead closely related taxa to exhibit neutral or negative relationships. Fourth, because the environments experienced by baboons in Amboseli are far more uniform than those experienced by typical human study subjects (Björk et al., 2022; Grieneisen et al., 2021), we expected that the signature of ‘universality’ in baboons would be stronger than that observed in humans. We discuss the implications of these patterns for individual microbiome community assembly and dynamics, and for understanding how microbiome communities are structured across hosts—a key requirement for successful intervention to improve host health (Bashan et al., 2016; Widder et al., 2016; Cullen et al., 2020).

## Results

### Most bacterial correlations within individuals are weak and negative

**Figure 2.**
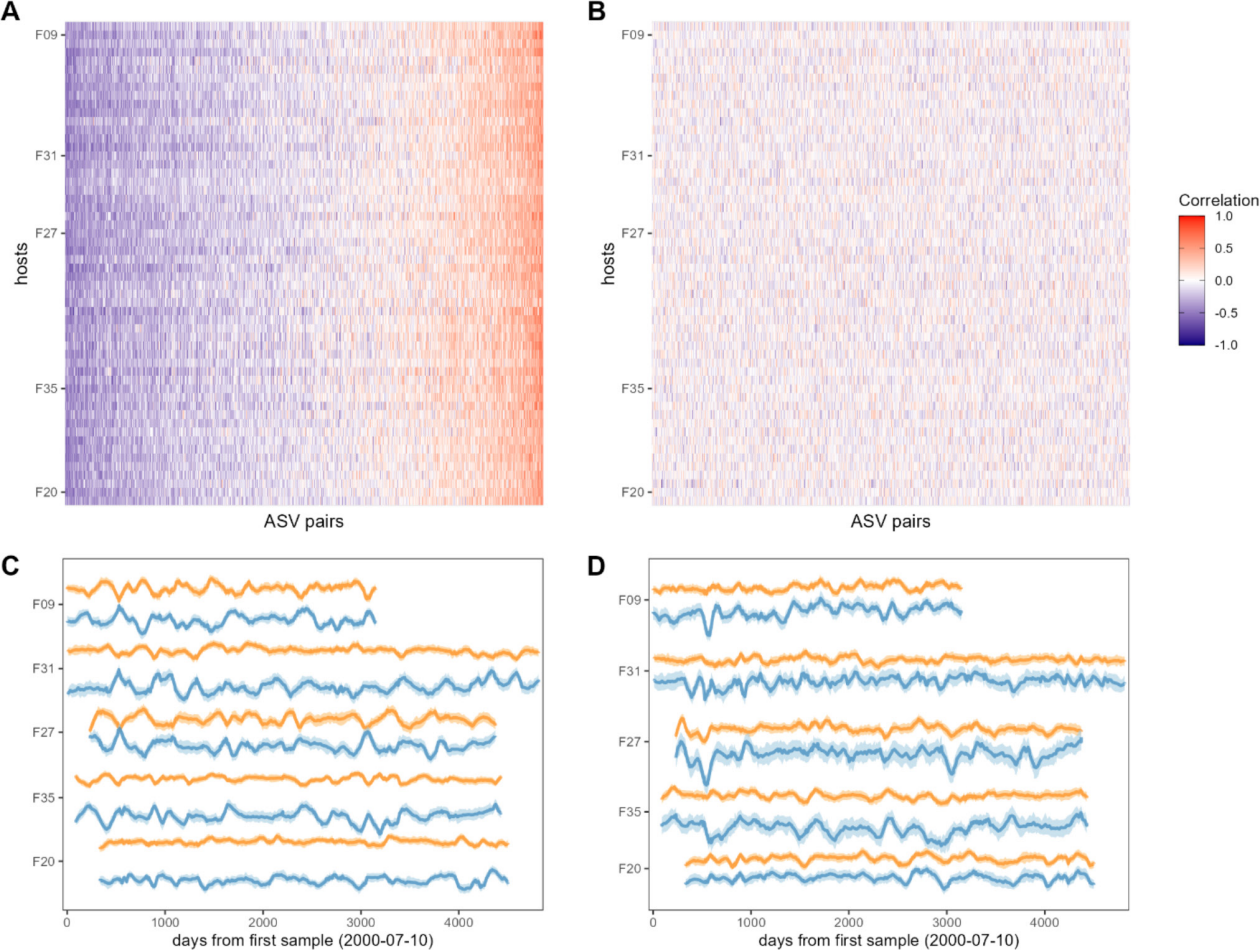
Bacterial correlation patterns across hosts. The heat map in panel (**A**) shows Pearson’s correlation coefficients of centered log-ratio (CLR) abundances between all pairs of amplicon sequence variants (ASVs) (*x*-axis) in each of the 56 baboon hosts (*y*-axis). Each pair of ASVs is represented on the *x*-axis, including all pairwise combinations of 107 ASVs with sufficient co-occurrence, resulting in 1878 ASV-ASV correlations measured per host (105,168 total correlations across all 56 hosts). Columns are ordered by the mean correlation coefficient between ASV-ASV pairs, from negative (blue) to positive (red). (**B**) Pairwise correlations generated from random permutations of the data. Taxonomic identities were shuffled within samples and pairwise ASV-ASV correlations were estimated to produce a null model of ASV-ASV correlation patterns within and between hosts. Column order is the same as in panel A. Panels (**C**) and (**D**) show example trajectories of CLR abundances for two pairs of ASVs in the same five hosts. Panel (**C**) shows a strongly negatively correlated pair (median *r* across all hosts = −0.461; two ASVs in order Clostridiales: ASV36 (orange) and ASV59 (blue); Supplementary file 1a and d) and panel (**D**) shows a strongly positively correlated pair (median *r* across all hosts = 0.508; two ASVs in genus *Bifidobacterium*; ASV1 (orange) and ASV4 (blue); Supplementary file 1a and d).

We began by characterizing the overall sign, strength, and significance of pairwise correlations in bacterial abundance within each host. To do so, we applied the approach outlined in Figure 1A to stool-associated time series from 56 baboons (Figure 1B) and calculated Pearson’s correlations between pairs of bacterial taxa. To avoid biases created by zero inflation (see Methods), we restricted our analysis to pairs where each member was present in at least 50% of samples across all hosts and at least 20% of samples within each host (Supplementary file 1a-1c). Further, we required the joint zero abundance of a given bacterial pair to be less than 5% of all samples across hosts. After filtering, the resulting data set included (1) 1878 pairs of centered log-ratio (CLR)-transformed amplicon sequence variants (ASVs; Figure 2A); (2) 57 pairs of bacterial phyla (Figure 2—figure supplement 1); and (3) 473 pairs of taxa agglomerated to the most granular possible family, order, or class (Figure 2—figure supplement 1). To generate an expectation of the strength of bacterial correlations possible by chance, we used a permutation procedure that randomly shuffled the taxonomic identities within each sample of the bacterial count table 10 times for each of the 56 hosts (560 total permutations). We then estimated correlations for these permuted pairs to generate an empirical null distribution of randomly generated taxon-taxon correlations. Observed correlations were judged against this reference (Figure 2B). We also confirmed that the resulting correlation patterns were robust to several modeling choices and were not primarily driven by seasonal shifts in microbiome composition (see results below).

**Figure 2—figure supplement 1.**
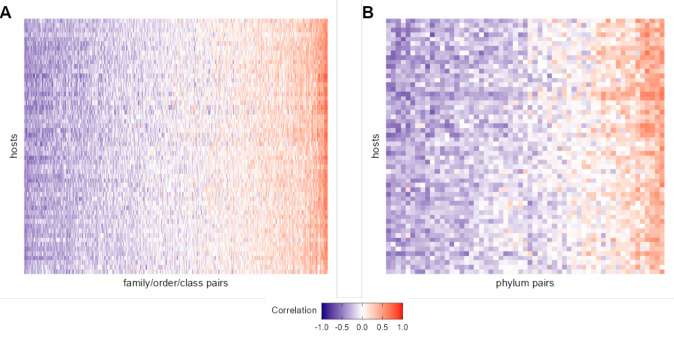
Heat maps of correlation of centered log-ratio (CLR) taxon pairs at taxonomic levels higher than amplicon sequence variant (ASV). Each heat map shows the Pearson’s correlation coefficient of CLR abundances between pairs of taxa that met our filtering criteria (*x*-axis) in each of the 56 baboons (*y*-axis). Panel (**A**) gives 473 pairs of taxa agglomerated to the most granular possible family, order, or class. Panel (**B**) gives 57 phylum-phylum pairs. See Figure 2A for a heat map of ASV-ASV correlation patterns across hosts.

**Figure 2—figure supplement 2.**
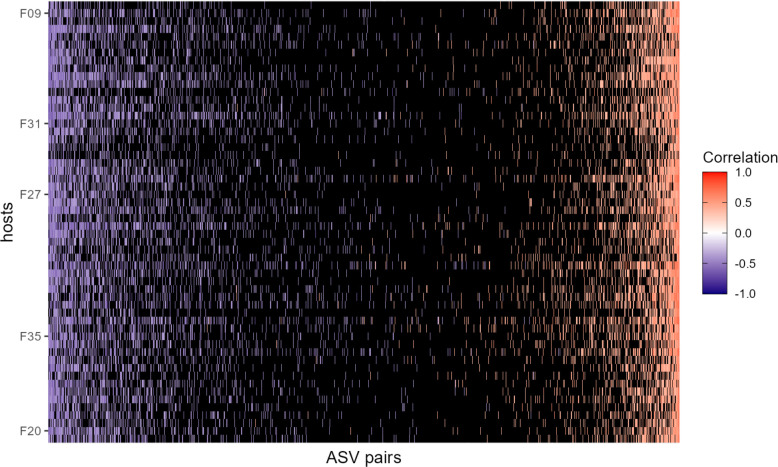
Heat map of correlation of amplicon sequence variant (ASV)-level centered log-ratio (CLR) taxon pairs where pairs not exceeding confidence cutoffs have been omitted. This heat map is identical to the one shown in Figure 2A in the main text, but black cells represent host-level ASV pairs whose estimated CLR correlation did not exceed the confidence cutoffs described in Figure 2—figure supplement 3, that is a CLR ASV-ASV correlation of less than –0.342 or greater than 0.328. The orientation of rows and columns is the same as in Figure 2A.

**Figure 2—figure supplement 3.**
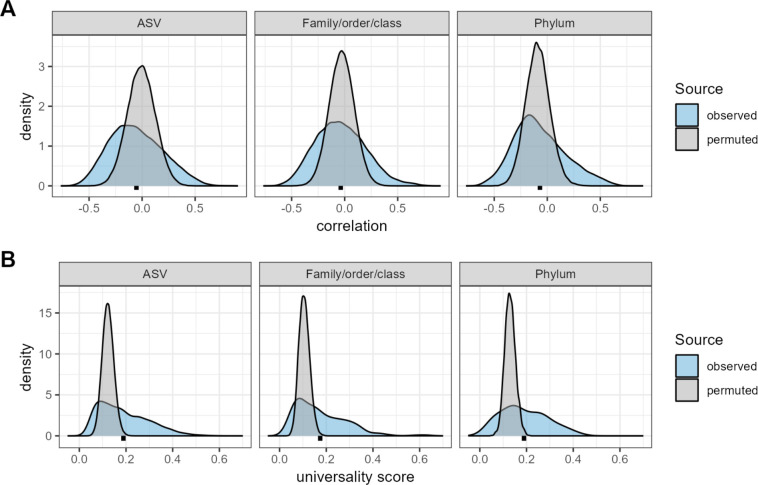
Confidence cutoffs for correlations and universality scores. The three plots in panel (**A**) show density distributions of the observed correlations between taxa (blue distributions) compared to random expectation (gray distributions) for three taxonomic partitions of the data: amplicon sequence variants (ASVs), family/order/class-level assignments, and phyla. The random distributions were generated by permuting the data 10 times per-host, shuffling taxonomic identities within individual microbiome samples and re-calculating taxon-taxon correlations in these permuted data sets. Mean correlations for the observed distributions are shown as black points below each distribution. The observed correlations for ASVs range from –0.767 to 0.904 (mean = –0.009; median = –0.010); for family/order/class designations range from –0.719 to 0.803 (mean = –0.028; median = –0.029); and for phyla from –0.645 to 0.687 (mean = –0.088; median = –0.092). Panel (**B**) shows density distributions of the observed universality scores in blue compared to random expectations in gray, for pairs of taxa at the ASVs, family/order/class-level, and phyla. Small, dashed black lines underneath each density indicate the mean observed universality score at that taxonomic level.

**Figure 2—figure supplement 4.**
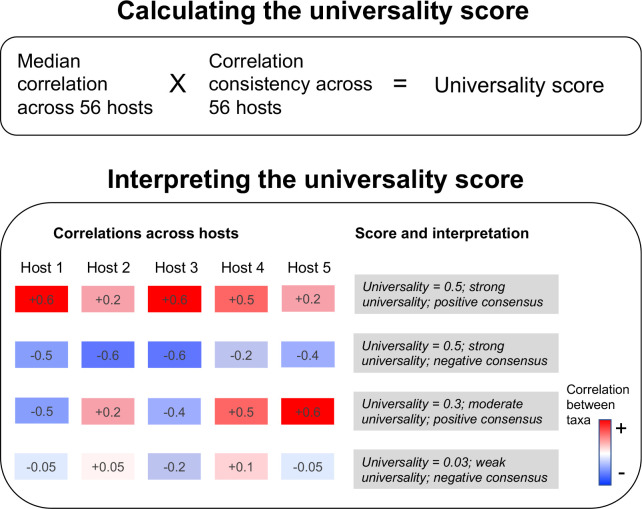
Our universality score identifies the bacterial pairs that exhibit the most consistent relationships across hosts. The score multiplies the pair’s median correlation coefficient across hosts with its correlation consistency across hosts (i.e. proportion of hosts in which the correlation has the same sign, either positive or negative). The resulting scores range from 0 to 1, where a score of 1 equates to perfect ‘universality’ (i.e. all hosts have a correlation coefficient of 1 or all hosts have a correlation coefficient of –1). The second panel illustrates the calculation of the score for 4 hypothetical taxa pairs across 5 hosts.

Consistent with the expectation that most bacterial correlations in the gut microbiome are weak (Coyte et al., 2015; Coyte et al., 2021), only 19% of ASV-ASV correlations in the heat map in Figure 2A were stronger than expected by chance (FDR≤0.05; Figure 2—figure supplement 3; 19% of phylum-phylum; 22% of family/order/class correlations; Figure 2—figure supplement 1; Figure 2—figure supplement 2). The strongest negatively correlated pair in Figure 2A included an ASV in the family Kiritimatiellae and another in family Lachnospiraceae which had a median correlation of –0.520 (±0.132 s.d.) across all baboon hosts (Figure 2C; ASV19 and ASV23; Supplementary file 1a and d). The strongest positively correlated pair of ASVs included two members of the genus *Prevotella* that had a median correlation of 0.801 (±0.053 s.d.) across all baboons (Figure 2D; ASV2 and ASV3; Supplementary file 1a and d). While these two ASVs were assigned to the same genus, their V4 16S DNA sequence identity was 97.6%, indicating they are probably not simply duplicate 16S gene copies encoded in the genome of a single species (Vetrovský and Baldrian, 2013 Supplementary file 1d).

In support of the idea that strong, positive bacterial interdependencies are rare (Coyte et al., 2015; Palmer and Foster, 2022; Coyte et al., 2021), only 3.8% of ASV pairs were significantly positively correlated, and the overall bacterial correlation patterns were slightly skewed toward negative relationships. For instance, at the ASV level, the median correlation coefficient in Figure 2A was –0.072, and 60% of these correlations were negative (binomial test p<0.0001). For family/order/class-level taxa, 58% of all correlations were negative (Figure 2—figure supplements 1A and 3A; median family/order/class-level correlation = −0.049; binomial test p<0.0001). Correlations between phyla exhibited the strongest negative skew, with 64% of phyla-phyla correlations having a negative sign (Figure 2—figure supplements 1B and 3A; median phyla-level correlation = −0.100; binomial test p<0.0001). This bias toward negative relationships is consistent with the expectation that neutral or negative relationships between ASVs are more common than mutualisms (Coyte et al., 2015; Palmer and Foster, 2022; Coyte et al., 2021) and that more distantly related taxa (e.g. phyla) respond to distinct environmental drivers due to differences in metabolic requirements and lifestyles.

### Within-host bacterial correlation patterns are largely consistent across baboons

**Figure 3.**
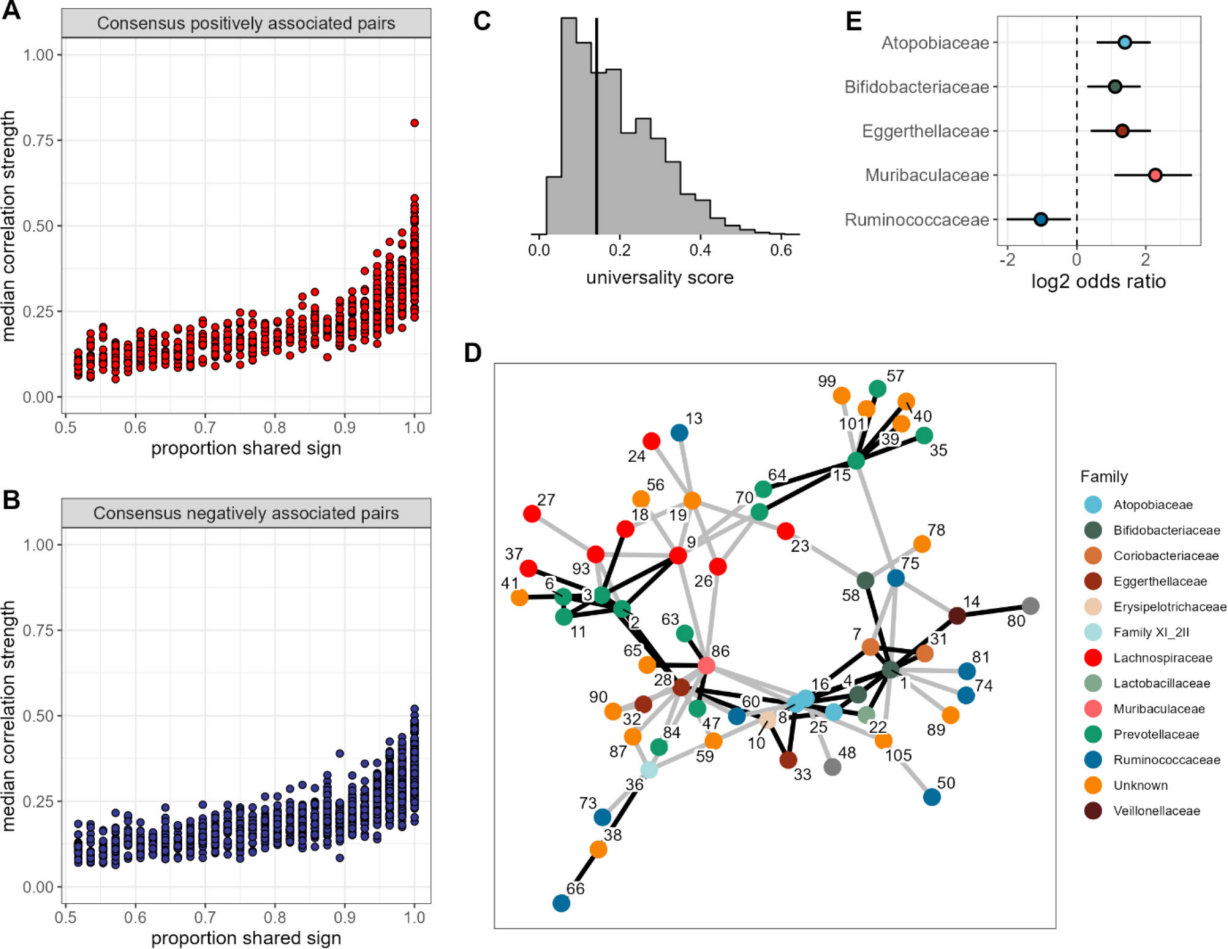
None of the amplicon sequence variant (ASV) pairs were strongly and inconsistently correlated across hosts, and the strongest and most consistently correlated ASVs are typically positively correlated. Plots in (**A**) and (**B**) show the median correlation strength for each ASV-ASV pair across all 56 hosts as a function of the consistency in direction of that pair’s correlation across hosts, measured as the proportion of hosts that shared the majority correlation sign (positive or negative; ASV pairs that were positively correlated in half of the 56 hosts have a consistency of 0.5; ASV pairs that were positively [or negatively] correlated in all hosts have a consistency of 1.0). Panel (**A**) presents this relationship for consensus positively correlated features and panel (**B**) shows consensus negatively correlated features. The Spearman’s correlation between median correlation strength and the proportion of shared sign for all correlated features is 0.844 (p<0.0001). Multiplying the two axes in either panel (**A**) or (**B**) creates a ‘universality score’ (Figure 2—figure supplement 4), whose distribution is shown in panel (**C**). This score reflects the strength and consistency of pairwise microbial correlations across hosts and ranges from 0 to 1, where a score of 1 indicates ASV-ASV pairs with perfect correlations of the same sign in all hosts. A vertical line indicates the minimum significant universality score. (**D**) Correlation networks for the top 5% most strongly and consistently correlated ASV pairs across hosts (i.e. the top 5% highest universality scores; pairs with rank 1–93 in Supplementary file 1d). Network edges are colored by the consensus sign of the correlation between that pair (black for pairs where most hosts had a positive correlation; gray for pairs where most hosts had a negative correlation). Node labels indicate the ASV identity in Supplementary file 1a and colors represent bacterial families. (**E**) Significantly enriched bacterial families in the network in panel D (Fisher’s exact test p<0.01 all, FDR≤0.05; see Supplementary file 1e enrichment statistics for all families).

Next, we tested the degree to which within-host ASV-ASV correlations were consistent across hosts. We began by plotting the absolute value of each ASV pair’s median Pearson’s correlation coefficient as a function of the consistency of their correlation sign (positive or negative) across the 56 hosts (Figure 3A and B). These plots provide two main insights into the consistency of bacterial associations. First, in support of the idea that ASVs do not exhibit vastly different correlative relationships in different hosts, no taxon pairs were strongly and *inconsistently* correlated across hosts (Figure 3A and B; Figure 3—figure supplement 1A). Instead, the ASV pairs that had inconsistent correlation signs across hosts always had weak and often non-significant median absolute correlation coefficients within hosts (Figure 3A and B).

**Figure 3—figure supplement 1.**
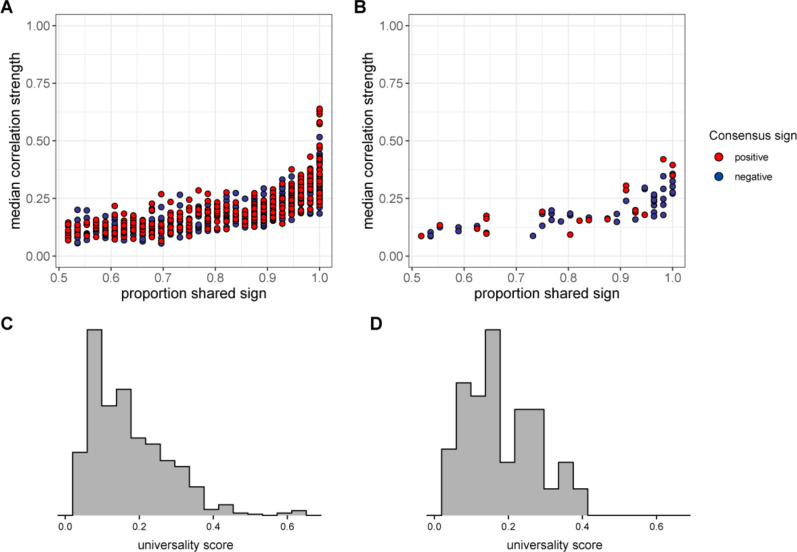
Universality at taxonomic levels higher than amplicon sequence variant (ASV). The plot in (**A**) shows the median absolute value of each family/order/class pair’s correlation coefficient across hosts as a function of correlation consistency, measured as the proportion of hosts that shared the majority correlation sign (positive or negative; taxon pairs that were positively correlated in half of the 56 hosts have a consistency of 0.5; pairs that were positively [or negatively] correlated in all hosts have a consistency of 1.0). Panel (**B**) plots the same estimates for phylum-phylum pairs. Multiplying the two axes in panels (**A**) and (**B**) creates a universality score reflecting the strength and consistency of pairwise microbial correlations across hosts. Universality score distributions for family/order/class pairs are shown in panel (**C**) and scores for phylum pairs are shown in panel (**D**).

**Figure 3—figure supplement 2.**
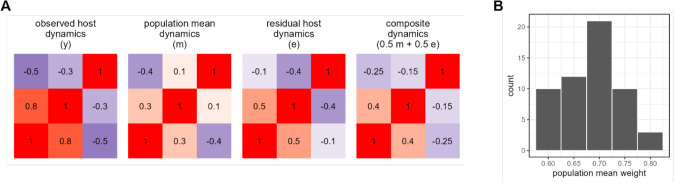
Quantifying the relative strength of universal versus individualized dynamics. Panel (**A**) shows observed (*y*), population mean (*m*), and host residual dynamics (*e* = *ym*) for a single hypothetical host with three amplicon sequence variants (ASVs). The fourth matrix shows a simulated case where host-level and population-level mean dynamics are added in equal proportions (0.5*m* + 0.5*e*) to approximate *y*. We estimate the proportion host- versus individual-level signal as the combination of mean and residual which best approximates *y*. Panel (**B**) shows the results of this procedure applied to the Amboseli data, giving a per-host estimate of population-level contribution to the observed centered log-ratio (CLR) ASV-ASV correlations.

Second, the pairs with the most consistent sign agreement across hosts also exhibited the largest median absolute correlation coefficients across hosts (Figure 3A and B; Spearman’s *r*=0.844, p<0.0001). Hence, pairs of ASVs that have the strongest relationships, and are therefore likely to play the most important roles in structuring gut microbiome dynamics, also tend to have the most consistent relationships in different hosts. Indeed, for the sets of positively or negatively correlated ASV pairs that showed universal agreement in the sign of their correlation across all hosts (i.e. where *x*=1 in Figure 3A and B), the median absolute correlation coefficient is 0.359, compared to 0.116 for those with no sign consistency (*x*=0.5 in Figure 3A and B). Note, that the correlation for a given pair of ASVs was only weakly predicted by bacterial abundance (*r*=0.129 and *r*=0.223 for the more and less abundant partner in a pair respectively; p<0.0001 both). While this effect was statistically significant, it explained only 6% of the variance in median correlation.

Visual inspection of the patterns in Figures 2A, 3A and B indicate that ASV-ASV correlations are largely consistent across baboons, as opposed to individualized to each baboon. To explicitly quantify the relative strength of shared versus individualized signatures in the heat map in Figure 2A, we calculated the population mean pattern for the ASV-ASV correlation matrix, *m*. For each host, we then estimated the residual difference, *e*, between that individual’s observed ASV-ASV correlation matrix, *y*, and the population mean matrix: *y – m* (see Figure 3—figure supplement 2A for a cartoon example). We reasoned that the observed correlation matrix for each host can be approximated by a mixture of contributions from the population mean matrix *m* and the host-specific residual matrix *e*. To identify the optimal mixture for each host (i.e. the mixture of consistent vs. individualized correlation patterns that best explained the observed data), we titrated the contribution (i.e. weight) of *e* from 0% to 100% (and correspondingly, the contribution of *m* from 100% to 0%) and identified the value that minimized the Frobenius distance between the simulated combination and the observed correlation matrix, *y*.

In support of prior observations of ‘universality’ (Bashan et al., 2016; Gao et al., 2020; Vila et al., 2020; San-Juan-Vergara et al., 2018), we found that, across hosts, the optimal mixture involved contributions from the shared correlation structure (i.e. *m*) of between 60% and 80% (median 70%) and a host-level contribution (i.e. from *e*) of between 40% and 20% (median 30%). Hence, population-level signatures contributed almost twice the weight as host-level signatures (a median population:host ratio of 2.33:1; Figure 3—figure supplement 2B). As a result, ASV-level relationships tend to be more consistent across hosts than host-specific.

### The most consistent ASV-level correlations are between phylogenetically related taxa

One advantage of our approach, compared to DOA (Bashan et al., 2016), is that we can identify the bacterial pairs that exhibit the most consistent relationships across hosts. Hence, we next conducted several analyses to understand why some taxon pairs are more consistent than others. To do so, we created a ‘universality’ score (Figure 2—figure supplement 4) that could be calculated for each ASV pair. The score multiplies the pair’s median absolute correlation coefficient across hosts (*y*-axis of Figure 3A and B) with its correlation consistency across hosts (i.e. proportion of shared sign; *x*-axis of Figure 3A and B). The resulting scores range from 0 to 1, where a score of 1 equates to perfect ‘universality’ (i.e. all hosts have a correlation coefficient of 1 or all hosts have a correlation coefficient of –1). Applying this score to all pairs of ASVs reveals a right-skewed distribution, reflecting the fact that most bacterial correlations are weak, with inconsistent sign directions across hosts (Figure 3C; Figure 2—figure supplement 3B). However, 48% of these scores were higher than expected by chance (permutation test; FDR≤0.05; Figure 3C; Figure 2—figure supplement 3B), reflecting a signal of universality in our data. Despite the bias toward negative ASV-ASV correlations in the overall set of bacterial correlations (Figure 2—figure supplement 3A), we observed no such bias in the most universal pairs. For instance, in the top 5% most universal ASV pairs (*n*=94 pairs), 46 pairs exhibited net positive correlations and 48 pairs, net negative correlations, suggesting no particular bias in the direction of the strongest and most consistent associations.

To visualize these highly consistent correlations, we plotted bacterial co-abundance networks connecting the top 5% most universal ASV pairs (Figure 3C). A handful of ASVs were highly connected within this network, especially ASV1 (genus *Bifidobacterium*; Supplementary file 1a and d), which was connected to 14 other ASVs. Three other ASVs were connected to at least 10 other ASVs, including members of families Atopobiaceae (ASV8), Eggerthellaceae (ASV28), and Muribaculaceae (ASV86) (Supplementary file 1e). These families were also enriched in this network, relative to the rest of the data, as was the family Bifidobacteriaceae (Figure 3E). Pairings between members of the same family were enriched by >3-fold in this network (p<0.0001), making up 32% of pairs in the most universal set versus only 9.8% of pairs outside that set. Almost two-thirds of these were Prevotellaceae-Prevotellaceae pairs (10 of 16 same-family pairs).

**Figure 4.**
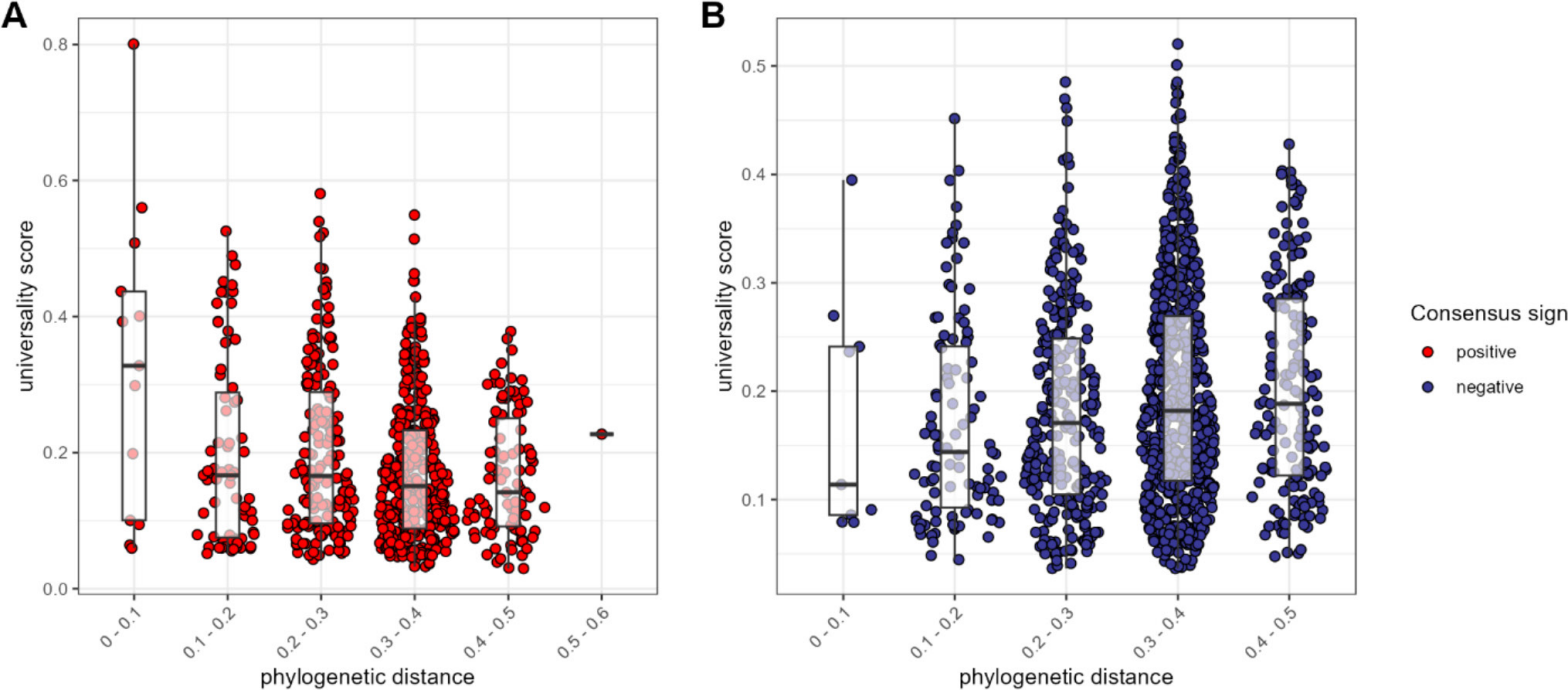
The most consistent amplicon sequence variant (ASV)-level correlations are positive and often between close evolutionary relatives. Pairwise universality scores are plotted as a function of phylogenetic distance between the ASV-ASV pair for consensus positively correlated pairs in red (**A**) and negatively correlated pairs in blue (**B**). Phylogenetic distance (*x*-axis) is binned into 0.1 increments; each point represents a given ASV pair, and box plots represent the median and interquartile ranges for a given interval of phylogenetic distance. Phylogenetic distance is negatively correlated with universality score in positive pairs (Pearson’s correlation for positively associated ASV pairs = −0.232, p-value <0.0001), and positively correlated with universality score in negatively associated pairs (Pearson’s correlation for negatively associated ASV pairs = 0.106, p=0.004). Full estimates are given in Supplementary file 1f.

We next asked: does the phylogenetic distance between a pair predict the nature of their relationship? In support of the idea that homology leads closely related ASVs to respond similarly to the environment, or perhaps facilitate each other’s growth (Meehan and Beiko, 2014; Vacca et al., 2020), we found that, for positively associated ASV pairs, closely related taxa had higher universality scores than more distantly related taxa (Pearson’s *r* for positively correlated pairs = −0.232; p<0.0001; Figure 4A). In contrast, when ASV pairs were negatively correlated, there was a weak positive relationship between phylogenetic distance and universality (Pearson’s *r*=0.106; p=0.004; Figure 4B). In other words, the strongest and most consistently *negatively* correlated taxa tend to be distantly related, whereas the strongest and most consistently positively correlated taxa were often closely related, especially members of the family Atopobiaceae (Supplementary file 1f).

**Figure 4—figure supplement 1.**
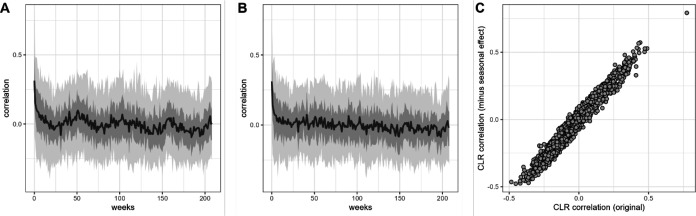
Estimates of centered log-ratio (CLR) ASV-ASV correlation are similar after removing seasonal trend. Panel (**A**) shows sample-sample autocorrelation estimates for the CLR-transformed data from 56 baboon hosts. Panel (**B**) shows sample-sample autocorrelation for the same data after the removal of a seasonal trend by arima() in R. Estimates of CLR ASV-ASV correlation derived from the original (basset) model and this per-ASV linear autoregressive model is highly similar, as shown in panel (**C**) where *r*=0.982. ASV, amplicon sequence variant.

**Figure 4—figure supplement 2.**
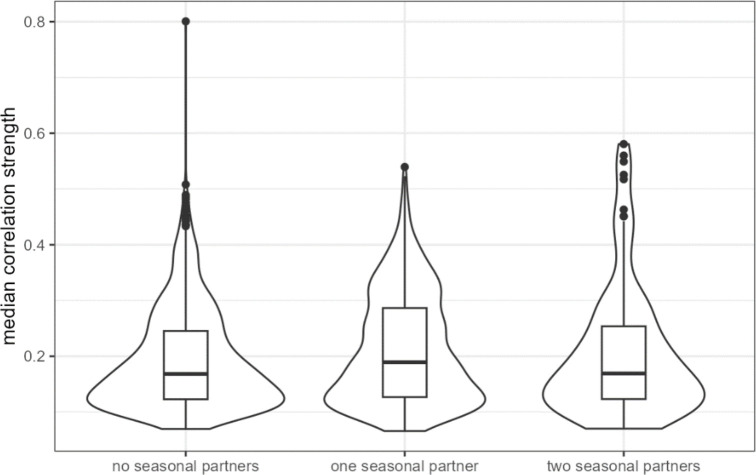
ASV-ASV pairs with two ‘seasonally varying’ partners have slightly higher median correlations across hosts. ‘Seasonally varying’ ASVs were those identified as having seasonally differential centered log-ratio (CLR) abundance in Björk et al., 2022. ASV-ASV pairs with at least one ‘seasonal’ member had significantly higher median correlation across hosts than did pairs where neither partner was seasonal (difference of 0.018, Wilcoxon rank sum test p = 0.000326). ASV, amplicon sequence variant.

**Figure 4—figure supplement 3.**
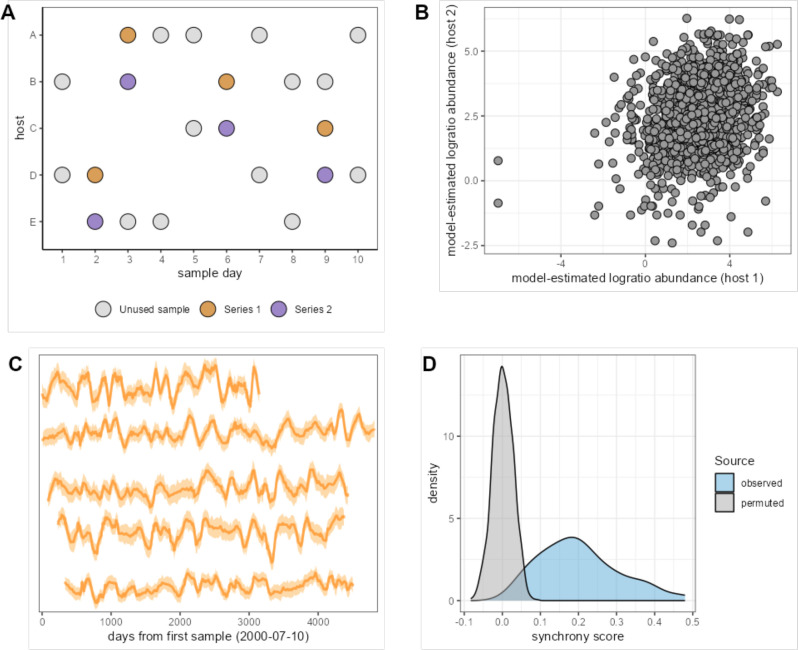
Measuring synchronized dynamics in the same amplicon sequence variant (ASV) across many hosts. The cartoon in panel (**A**) shows the sample selection procedure. We selected pairs of samples from different hosts collected within 24 hr of each other, producing 20,000 ‘same-day’ pairs (e.g. purple and mustard-colored pairs on days 2, 3, 6, and 9). We subset these pairs to one same-day pair per host pair, producing 1540 same-day pairs from these thinned host series and extracted the correlated ASV abundance from these pairs. Synchrony for a given taxon is estimated as the correlation of the centered log-ratio abundance of that taxon across Series 1 and 2. Panel (**B**) shows the correlation between log-ratio abundance estimates in paired host samples for the most synchronous taxon, ASV #21 (Clostridiaceae 1; *r* = 0.480). Panel (**C**) shows the time-aligned model-estimated centered log-ratio abundances of ASV #23 in five well-sampled hosts that each lived in a different baboon social group (hosts from top to bottom are F09, F31, F35, F27, F20). Panel (**D**) shows the distribution of observed synchrony estimates for all 125 ASVs (light blue) compared to synchrony estimates from a permuted/null distribution (gray); 118 of 125 ASVs had significantly higher synchrony than expected (FDR≤ 0.05; permutation scheme).

**Figure 4—figure supplement 4.**
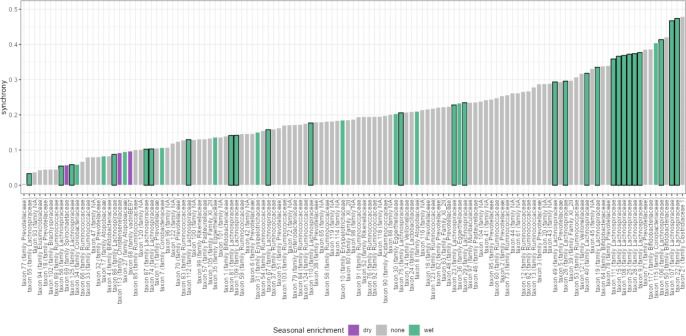
Seasonality is not associated with synchrony. Synchrony estimates for all amplicon sequence variants (ASVs) are given as bar plots. ASVs that were identified as having seasonally differential centered log-ratio (CLR) abundance in Björk et al., 2022, are labeled by the season in which they were more abundant: dry or wet. Seasonally variable labels do not predict synchrony (ANOVA, p=0.358).

**Figure 4—figure supplement 5.**
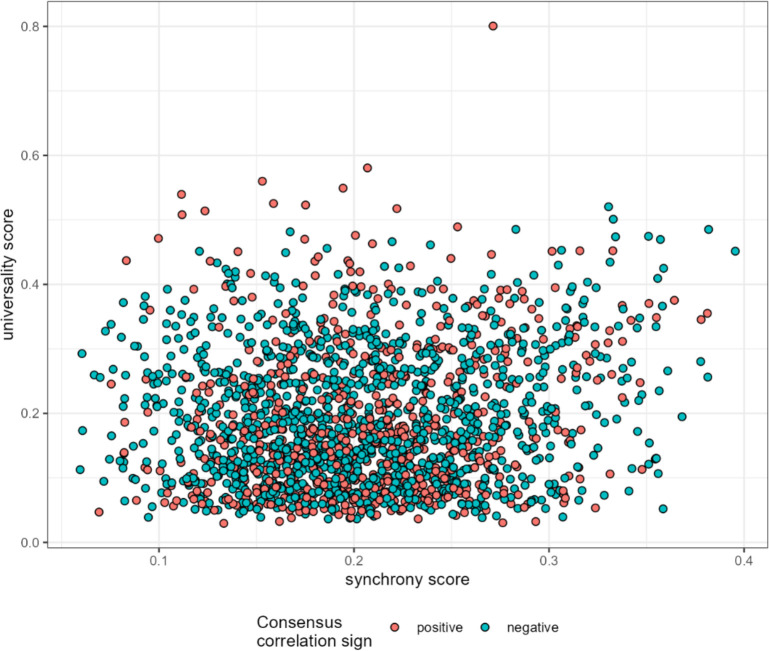
Synchrony weakly predicts universality. Mean ASV-ASV pair synchrony estimates are plotted against universality scores for all 1878 high-confidence ASV pairs. Synchrony weakly predicts universality (*r*=0.116, p<0.0001). ASV, amplicon sequence variant.

### Genetic relatives, hosts with similar microbiome compositions, and age mates have more similar bacterial correlation patterns

**Figure 5.**
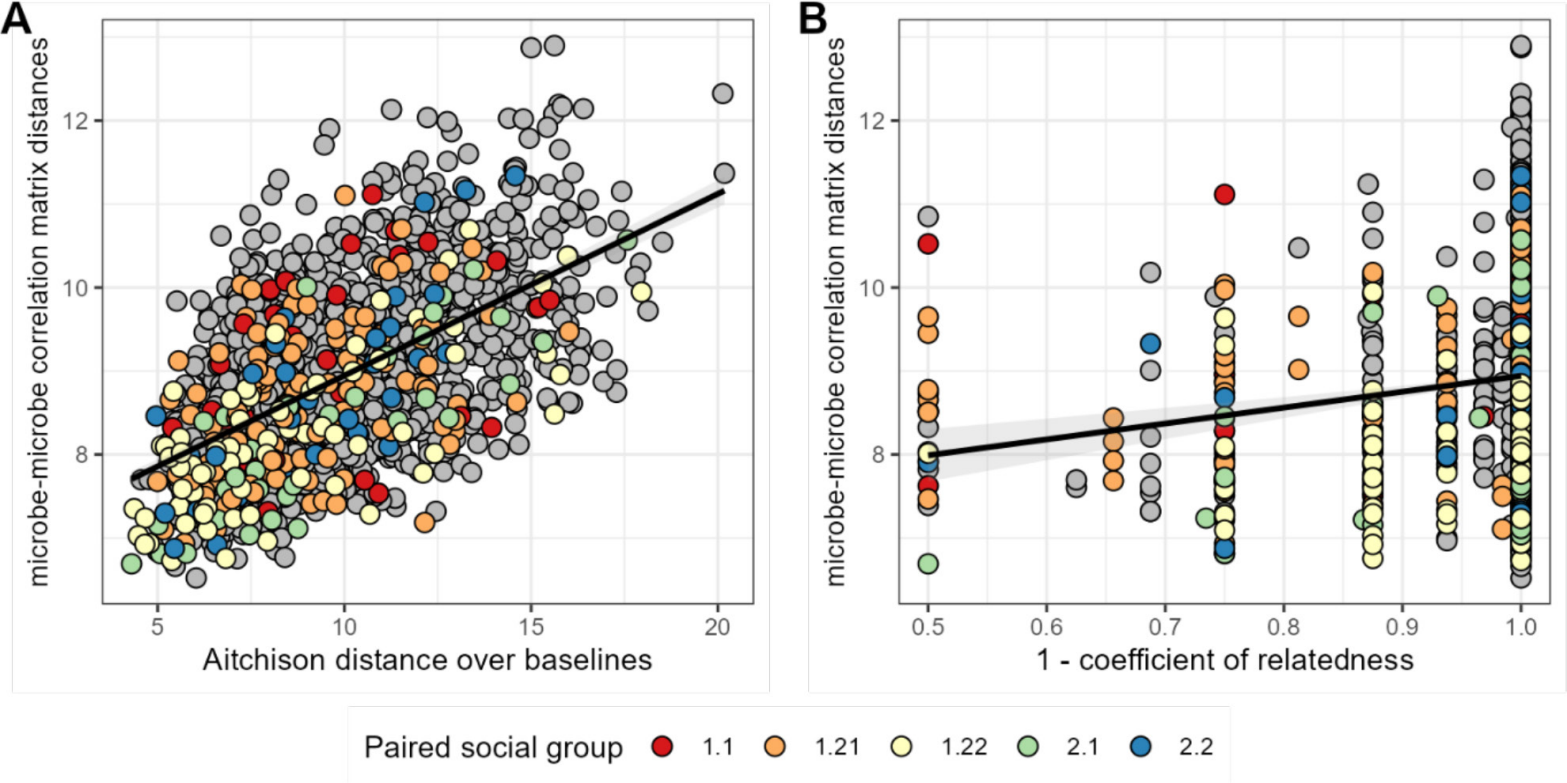
Baboons with more similar bacterial correlation patterns are more likely to have more similar baseline microbiome compositions and are slightly more likely to be genetic relatives. In panel (**A**) each point is a pair of hosts; the *y*-axis shows the similarity of these hosts’ bacterial correlation patterns (via Frobenius distance) as a function of their microbiome compositional similarity (via Aitchison distance; Mantel: *r*^2^=0.336; p=0.001). Colors show samples from pairs of baboons living in the same social group and gray dots are pairs of animals living in different social groups. There is no detectable effect of social group on correlation pattern similarity. Panel (**B**) shows the same distances as a function of host genetic dissimilarity (1 – the coefficient of genetic relatedness between hosts; *r*^2^=0.009; p-value Mantel test 0.002). Colors reflect pairs of hosts living in the same social group, as in panel A.

We next asked whether host attributes, including host sex, social group membership, genetic relatedness, age, or baseline gut microbiome composition, predict host differences in patterns of bacterial correlation. Consistent with studies that use DOA to infer universality (Bashan et al., 2016; Gao et al., 2020; Vila et al., 2020; San-Juan-Vergara et al., 2018; Kalyuzhny et al., 2017), the strongest predictor of distance in bacterial correlation patterns was distance in terms of baseline microbiome composition (a core assumption of DOA). Indeed, a Mantel test correlating compositional distance of average microbial profiles (as Aitchison distances between the per-host mean of CLR-transformed samples) with distance in microbial correlation patterns between hosts revealed that 34% of the variation in correlation patterns was explained by baseline microbiome community composition (Mantel: *r*^2^=0.336; p=0.001; Figure 5A; Supplementary file 1g).

**Figure 5—figure supplement 1.**
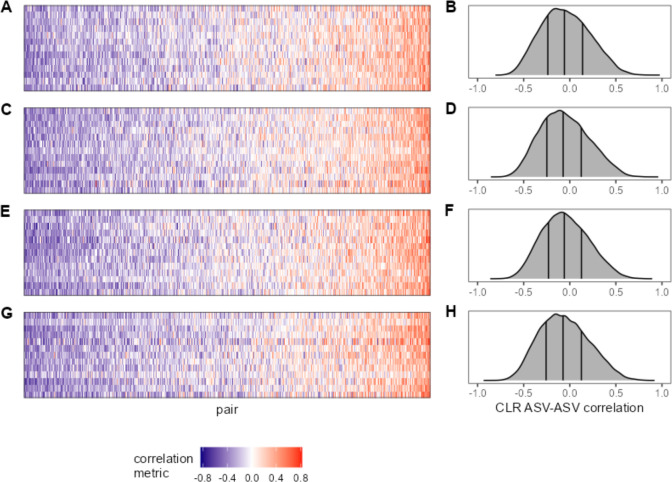
Within- and between-age bin ASV-ASV correlations are similar. For a subset of hosts with sufficient sampling across age ‘bins’ (at least 35 samples within two of the following age ranges: juvenile = 0–6 years; prime age adult = 6–13 years; older adult = 13+ years), ASV-ASV correlation is visualized as a heat map. Panel (**A**) shows ‘juvenile’ ASV-ASV correlations for hosts F32, F06, F27, F36, F10, F01, F07, F35, M09, F05, M10, F22, F26 (top row to bottom) and (**B**) the distribution of those correlations. Panels (**C**) and (**D**) give ‘prime age’ correlations for the same hosts. Panel (**E**) shows prime age ASV-ASV correlations for the hosts F28, F17, F16, F31, M03, M08, F04, F12, F03, F33, M02, F14, F08 (top row to bottom) and (**F**) the distribution of those correlations. Panels (**G**) and (**F**) give ‘older adult’ correlations for the same hosts. ASV, amplicon sequence variant.

**Figure 5—figure supplement 2.**
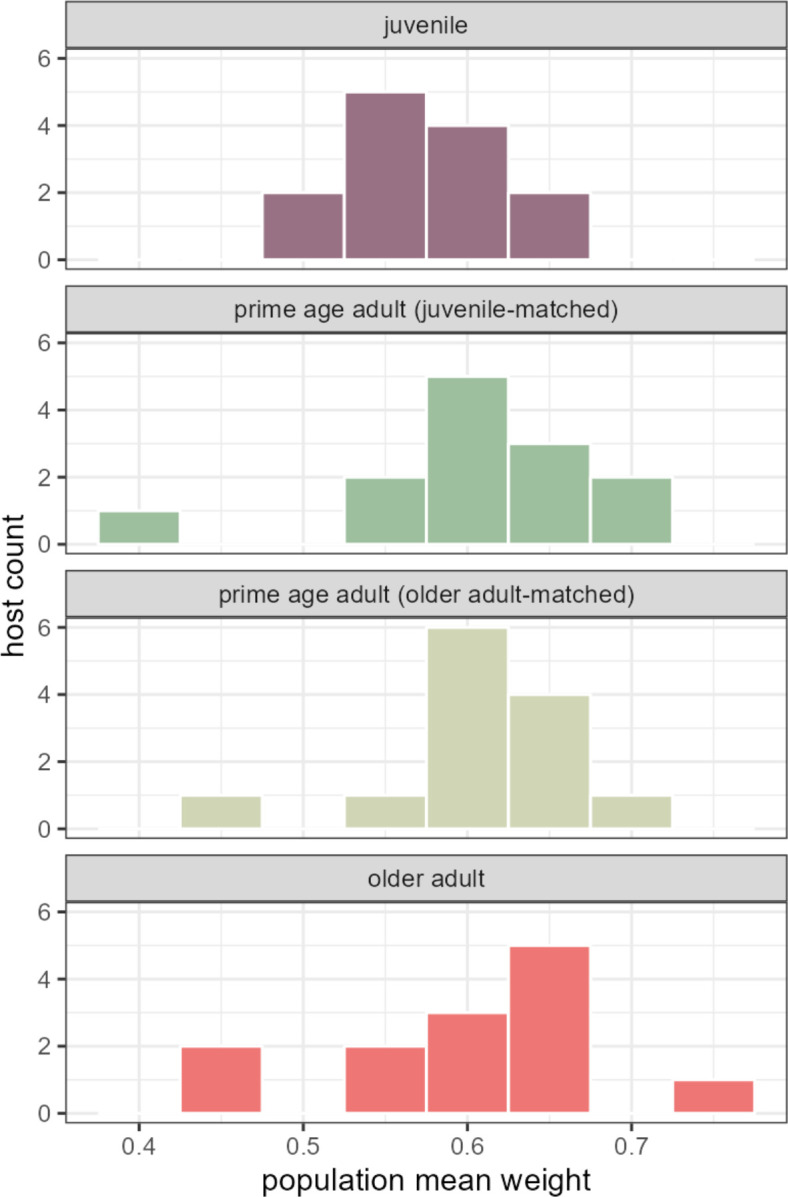
Quantifying the relative strength of universal versus individualized dynamics across age ranges. Histograms show estimates of the population-level contribution to observed centered log-ratio (CLR) ASV-ASV correlations in each 13-host age bin. (Hosts are matched between age bins where noted.) Variation in the population-level contribution to observed CLR ASV-ASV correlations is slightly, but not significantly, larger in the prime age and older adult bins than in the juvenile bin. Compare these estimates to those obtained for overall hosts series in Figure 3—figure supplement 2. ASV, amplicon sequence variant.

**Figure 5—figure supplement 3.**
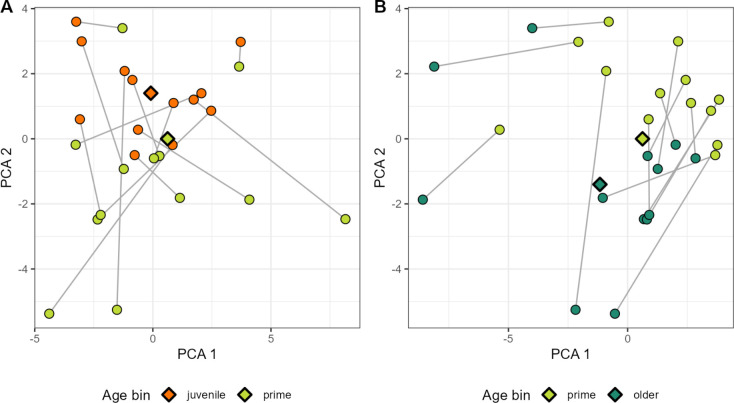
PCA plot of ASV-ASV correlations within age bins for a select set of hosts. For a subset of hosts with sufficient sampling across age ‘bins’ (at least 35 samples within two of the following age ranges: juvenile = 0–6 years; prime age adult = 6–13 years; older adult = 13+ years), ASV-ASV correlation is visualized along principal components (PCs). Circles indicate the set of correlation estimates for a particular host within a particular age bin. Circles in panel (**A**) refer to ‘juvenile’ and ‘prime age’ hosts in orange and green; panel (**B**) refers to ‘prime age’ and older adults in light and dark green. Estimates across age bins (e.g. juvenile vs. prime age) for the same host are linked by a gray line. Diamonds indicate the centroid for each age bin. For the juvenile vs. prime age comparison in panel A, the ASV pair contributing most strongly to variation along PC1 was a genus *Libanicoccus*-genus *Bifidobacterium* pair (ASV16-ASV58). The pair contributing most strongly to placement along PC2 was a genus *Lactobacillus*-order Clostridiales pair (ASV22-ASV36). In the prime age adult vs. older adult embedding in panel B, the ASV pair which contributed the most to variation along PC1 was a genus *Prevotella 9*-unknown bacterium pair (ASV2-ASV40). The pair with the greatest contribution to PC2 was another taxonomically unresolved bacterium (ASV41) in combination with another member of family Prevotellaceae (ASV64). ASV pairs with the largest loadings in PCs 1 and 2 for both age ranges are given in Table S9. ASV, amplicon sequence variant.

Consistent with prior research in our population, which finds widespread heritability for gut microbiome taxon abundances (Grieneisen et al., 2021), we also found a weak but significant relationship between host genetic distance and the distance in microbial correlation patterns between hosts, after controlling for similarity of baseline composition across hosts. Hosts who were more closely related based on a multigenerational pedigree have slightly more similar ASV-level correlation matrices (Figure 5B; Supplementary file 1g; *r*^2^=0.009; partial Mantel controlling for baseline similarity: p-value = 0.002). We found no evidence that members of the same social group or sex exhibit more similar microbial correlation patterns (social group: F=2.146; p=0.089; sex: F=2.026; p=0.160; Supplementary file 1g).

Host age may also predict the overall strength of microbial relationships, and some studies find that gut microbial compositions become more individualized with age (Risely et al., 2022; Wilmanski et al., 2021). This observation suggests that host age may also be linked to individualized microbial relationships. To test these ideas, we divided our hosts into three classes: juveniles (0–6 years), prime age adults (6–13 years), and older adults (13+ years) and compared ASV correlation patterns between (1) the juvenile and prime age class and (2) between prime age and older adults. Hosts were only included in these analyses if they had >35 samples in either the juvenile and prime age class or prime age and older adult class (no host had >35 samples in all three age classes; 13 hosts were included in the juvenile and matched prime age adult groups; a separate set of 13 hosts were included in the older adult and matched prime age adult groups).

We found no evidence that microbial correlations get stronger or weaker with age (Figure 5—figure supplement 1). Further, using the same methods described above to estimate the relative host- and population-level contributions to ASV correlation patterns, we found no strong differences in the degree of ‘personalized’ correlation patterns across age groups (Figure 5—figure supplement 2). Further, ASV correlation patterns were slightly more similar within versus between adjacent age classes (juvenile vs. prime age, ANOVA p=0.00175, 1.3% variance explained; prime age vs. older adult, ANOVA p=0.0112, 0.9% variance explained). Principal components analysis on the microbial correlation patterns between juvenile and prime age hosts, and between prime age and older adult hosts revealed some age effects, particularly on the second principal component (Figure 5—figure supplement 3). See the legend of Figure 5—figure supplement 3 and Supplementary file 1i for information on which ASV pairs differed across age categories.

### Universality in Amboseli is not well explained by microbes’ shared responses to diet, season, or synchronized dynamics

Without experiments, we cannot disentangle whether our observed bacterial correlations are due to ecological interactions between bacterial species or to shared responses to environmental gradients, either inside or outside the host. While we were unable to control for many aspects of the host environment (e.g. gut pH, hormones, or immune profiles), we were able to include measures of dietary variation in our models of microbial abundances. Seasonal changes in host diet therefore do not account for universality in microbial relationships across hosts.

To account for additional unexplained seasonal variation, we next removed the oscillating seasonal trend from the log-ratio abundances for each ASV (modeled as a sine wave) and re-estimated the ASV-ASV correlation matrix (Figure 4—figure supplement 1). Removing the seasonal trend had little effect on ASV-ASV correlations, as the variance explained by seasonal oscillation was small for all ASVs (median 1.1%, minimum = 0%, maximum = 6%). Consequently, the between-ASV correlation estimates were almost identical to those derived from our original model (Pearson’s *r*=0.982, p<0.0001; Figure 4—figure supplement 1C). Further, ASV pairs where one or more members was from a family that showed strong seasonal changes in a prior analysis of these data (Björk et al., 2022), henceforth ‘seasonal’ families, had only slightly higher universality scores than taxon pairs where neither partner showed strong seasonal changes in abundance (difference of 0.018; p<0.0001; Figure 4—figure supplement 2).

Because the high level of universality we observed was not well explained by season, we also tested whether universality was explained by synchronized dynamics. We reasoned that if one member of an ASV pair shows highly synchronized dynamics across different hosts, and the other member is also strongly synchronized across hosts, then universality could be an inevitable outcome of strong, but independent synchrony in both members of the pair. We quantified synchrony as the degree to which the observed dynamics of a single, focal ASV are consistent across hosts, such that high synchrony (near 1) implies that the timing and direction of shifts in log-ratio ASV abundance are identical across hosts in the population (see Methods; Figure 4—figure supplement 3). Estimates of synchrony ranged from 0.033 to 0.474 (median=0.187). Interestingly, ASVs in the 13 ‘seasonal’ families are not more likely to have high synchrony than other families (ANOVA, p=0.434; Figure 4—figure supplement 4; Supplementary file 1h). The average synchrony of an ASV-ASV pair had a statistically significant but weak relationship with that pair’s universality score (*r*=0.116, p<0.0001; Figure 4—figure supplement 5).

### Baboon microbiomes are not substantially more ‘universal’ than human microbiomes

Finally, to investigate parallels between baboon and human microbial communities, we turned to two publicly available gut microbial time series data sets: daily samples from 34 human adults over a 17-day span (483 total samples; hereafter Johnson et al., 2019), and the DIABIMMUNE cohort that consists of 285 samples, collected monthly over 3 years, from 15 infants and toddlers living in Russian Karelia (Vatanen et al., 2016; at the time of writing, these cohorts were the only publicly available data sets we could find that included large numbers of repeated samples from the same subjects). Because baboons in Amboseli experience less heterogeneity in their environments and diets than humans (Björk et al., 2022; Grieneisen et al., 2021), we expected they would exhibit greater consistency in microbial correlations than either human cohort. Here, we compared each host cohort’s universality at the level of correlations between families, orders, and classes because these taxonomic levels offered the greatest comparative power (10.1% of families/orders/classes overlap between the cohorts compared to just 3.1% of genera and no ASVs).

**Figure 6.**
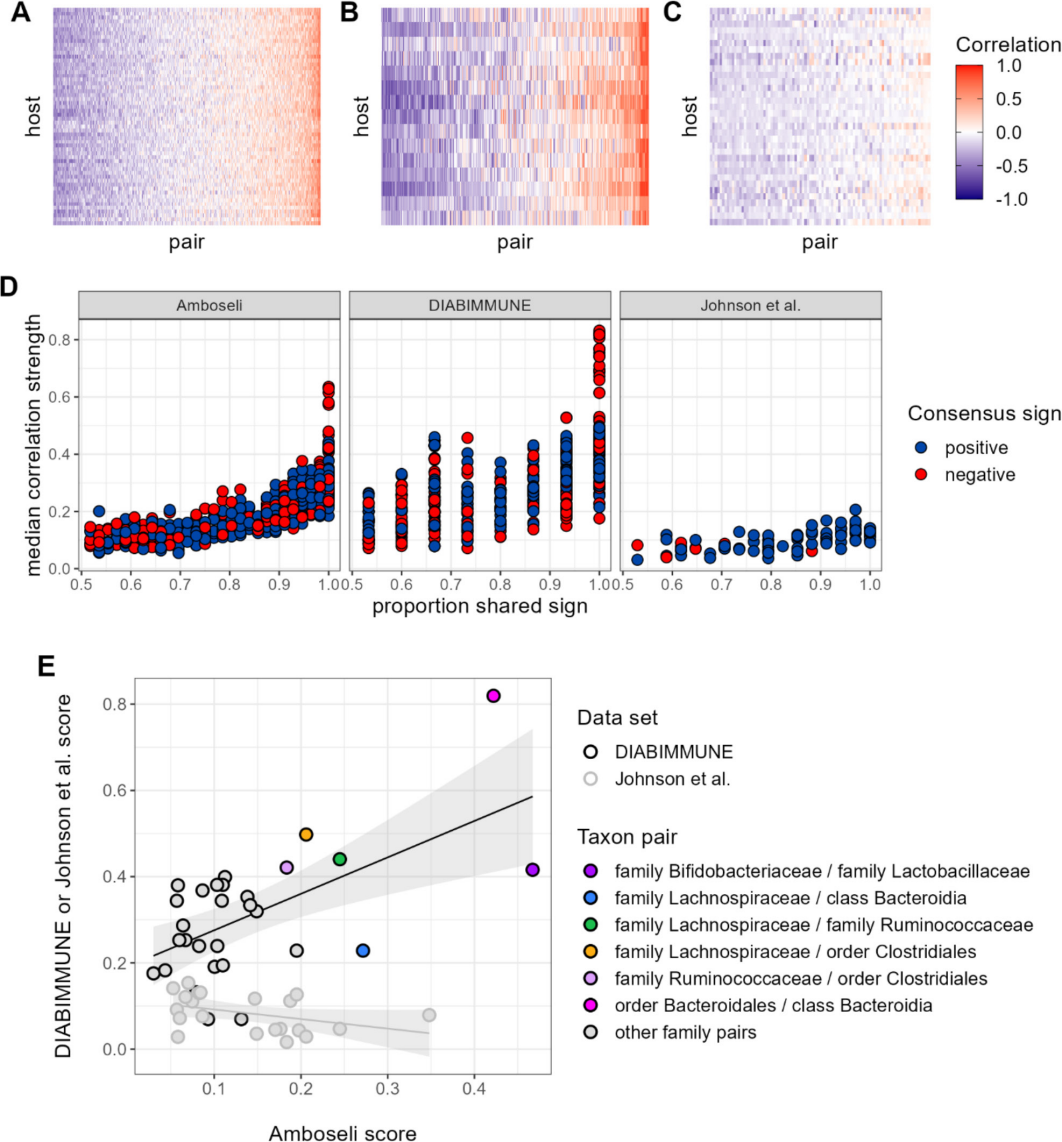
Patterns of universality in baboons are recapitulated in the DIABIMMUNE study. Following Figure 2A, panels (**A**), (**B**), and (**C**) show the Pearson’s correlation coefficients of centered log-ratio (CLR) abundances between all pairs of families (*x*-axis) in two time series data sets from human subjects: (**A**) the Amboseli baboons, (**B**) the DIABIMMUNE cohort, consisting of 15 infants/toddlers sampled monthly over 3 years in Russian Karelia (Vatanen et al., 2016), and (**C**) the diet study of Johnson et al., 2019, including 34 adults sampled daily over 17 days. Following Figure 3A and B, panel (**D**) shows the median correlation strength of each family pair’s correlation coefficient across hosts as a function of the consistency in direction of that pair’s correlation across hosts (i.e. the proportion of hosts that shared the majority correlation sign, positive or negative). Median correlation strength is low overall in Johnson et al. (median = 0.090), whereas the Amboseli baboon and DIABIMMUNE infant/toddler cohorts show similar relationships between median correlation strength and the proportion shared correlation sign across hosts (Spearman’s *r* in Amboseli = 0.844; Spearman’s *r* in DIABIMMUNE = 0.690). (**E**) Universality scores for overlapping family pairs from the infant/toddler subjects of the DIABIMMUNE study and baboons in the Amboseli study are significantly correlated (*r*=0.784, p=0.00161). Black outlined points are family pairs that overlapped between the Amboseli baboon and DIABIMMUNE infant/toddler data sets; gray outlined points are family pairs that overlapped between the Amboseli and Johnson et al. data sets. Color represents the taxonomic identities of the family pairs.

Contrary to our expectations, we find comparable evidence of universality in baboons and the DIABIMMUNE infant/toddler cohort, but weaker evidence for universality in Johnson et al. (Figure 6A–D). Bacterial families in the DIABIMMUNE cohort yielded universality scores slightly higher than those observed in Amboseli (25th percentile=0.142, median=0.216, 75th percentile=0.321 for DIABIMMUNE; 25th percentile=0.088, median=0.150, 75th percentile=0.234 for Amboseli), driven by correlations between families that were stronger on average than those estimated for baboons (median DIABIMMUNE family-family correlation strength=0.253; median Amboseli family-family correlation strength=0.170). The high level of consistency between both human infants/toddlers and wild baboons is surprising and may be due to the similar sampling intervals for these cohorts. Both cohorts were sampled approximately monthly, while Johnson et al.’s subjects were sampled daily (Coyte et al., 2021; Guittar et al., 2019). Median correlation strengths and universality scores for the Johnson et al., 2019, cohort were substantially lower (median correlation=0.090; 25th percentile universality=0.050, median=0.086, 75th percentile=0.113) than the DIABIMMUNE cohort or the baboons.

Despite considerable differences in the hosts, time scales, and designs of these studies, all three data sets exhibited a positive correlation between correlation strength and sign consistency for family pairs (Figure 6D). This trend was strongest in the Amboseli baboons (exponent of power regression *b*=1.72; p<0.0001); weaker in the DIABIMMUNE cohort (*b*=1.51; p<0.0001) and weakest in Johnson et al., 2019 (*b*=1.19; p<0.0001). Further, the most universal family-family associations skewed positive in both the baboons and the infant data set. All of the top 5% most universal family pairs (30 of 30 pairs) are positively associated in the DIABIMMUNE cohort, compared to 70% (23 of 33 pairs) in the Amboseli baboons.

Finally, we examined the relationship between universality scores for family pairs that overlapped between Amboseli and DIABIMMUNE (n=29 pairs), and between Amboseli and Johnson et al., 2019 (Figure 6E; n=21 pairs; only 10 family pairs overlapped between all three data sets). For these overlapping pairs, scores in the Amboseli data predicted scores for the same family-family pair in the DIABIMMUNE data set (*r*=0.562, p=0.001). The association between scores in the Amboseli data and the Johnson et al. data was negative, but not statistically significant (*r*=−0.402, p=0.071).

## Discussion

Do different hosts have different microbiome ‘ecologies’? Ecological and evolutionary processes like horizontal gene flow, genotype by environment interactions, and priority effects have been predicted to lead bacterial species to occupy different niches (with different ecological interactions) in different communities (Dolinšek et al., 2016; Franzosa et al., 2015; Faith et al., 2013; Bik et al., 2016; Caporaso et al., 2011; Costello et al., 2009; Louca et al., 2018; Rainey and Quistad, 2020; Martiny et al., 2015). Yet contrary to these expectations, here we find that hosts in the same population exhibit pairwise bacterial correlation patterns that are predominantly shared across hosts, rather than idiosyncratic to individual hosts. If these shared correlation patterns arise from shared microbiome ecologies, this discovery has consequences for understanding the basic eco-evolutionary drivers of microbiome dynamics and for human and animal health. For instance, shared ecologies would mean that designing widely applicable microbiome interventions is a more attainable goal than personalized microbiome compositions would suggest. Shared microbiome ecologies may also enable researchers to develop microbiome interventions that leverage these interactions to manipulate the microbiome’s emergent community properties to improve host health.

By measuring bacterial correlations in multiple hosts, we were also able, for the first time, to pinpoint which pairs of bacterial taxa exhibit the most consistent relationships across hosts. We found that most bacterial abundance correlations—from ASV-ASV to phyla-phyla relationships—were weak and negative. Positive bacterial interactions have been the subject of recent discussion in the literature (Loftus et al., 2021; Palmer and Foster, 2022; Kehe et al., 2021). Ecological theory predicts that strong positive interactions should be rare in natural communities because species interdependencies can hamper community assembly and stability (Coyte et al., 2015; Coyte et al., 2021). This theory is supported by experiments that directly measure the effects of one bacterial species on another’s growth (Weiss et al., 2022; Ortiz et al., 2021; Carlström et al., 2019; Venturelli et al., 2018) (but see Kehe et al., 2021). Our results suggest that strong, positive bacterial correlations are indeed uncommon in intact, unmanipulated microbiomes: significant positive relationships made up just 3.8% of all the pairwise correlations we observed. Hence, strong mutualisms, while key to microbiome function and dynamics, are probably rare in gut communities.

While mutualisms and universal dynamics are important, the correlation patterns we observe likely arise from a combination of ecological interactions between bacteria and shared responses to the environment (i.e. pairs of bacteria that prefer the same or different environments). In support of the idea that at least some of the correlations we observed are due to between-species interactions, our signature of universality was essentially unchanged after accounting for some of the strongest known drivers of microbiome composition and change in our population—host diet and season (Björk et al., 2022; Grieneisen et al., 2021)—as well as microbial synchrony between hosts. However, our approach did not account for important environmental gradients within the gut, such as host immune profiles and intestinal pH. These factors also shape microbiome composition (e.g. Reese et al., 2018; Firrman et al., 2022; de Vos et al., 2022), and can lead to shared abundance correlations between hosts even if hosts themselves differ. Ecological selection via within-host environments may explain our finding that genetic relatives share somewhat similar bacterial correlation patterns. Ecological selection is also consistent with our observation that the most consistent ASV-level correlations are between phylogenetically related taxa, and these patterns were strongest for positively associated taxon pairs. In support, phylogenetically related species have been shown to have similar environmental preferences (Tamames et al., 2016). We note that none of the correlations we observed can be mapped directly to standard categories of pairwise ecological interactions, such as mutualism, commensalism, amensalism, exploitation, or competition. Experimental approaches that directly measure the effects of one species on another’s growth in vitro are better suited to characterizing these relationships (Kehe et al., 2021; Weiss et al., 2022; Ortiz et al., 2021; Carlström et al., 2019; Venturelli et al., 2018).

The strong signal of universality we observed in bacterial abundance correlations stands in contrast to the common observation that microbiome taxonomic composition (i.e. the presence and abundance of bacterial species) is almost always highly personalized (Franzosa et al., 2015; Faith et al., 2013; Bik et al., 2016; Caporaso et al., 2011; Costello et al., 2009; Risely et al., 2021; Kolodny et al., 2019; Flores et al., 2014; Johnson et al., 2019; Pruss et al., 2021). Our own prior analyses of these data found that each baboon exhibited personalized microbiome compositions and asynchronous single-taxon dynamics (Björk et al., 2022). These contrasting patterns—personalized compositions but shared abundance correlations—are important because personalized microbiota have been proposed to arise, at least in part, from personalized microbiome ecologies (Franzosa et al., 2015; Faith et al., 2013; Bik et al., 2016; Caporaso et al., 2011; Costello et al., 2009; Risely et al., 2021; Kolodny et al., 2019; Flores et al., 2014; Johnson et al., 2019; Pruss et al., 2021). We can think of at least three explanations that reconcile these observations. First, consistent with ideas discussed above, if environments in the gut shape bacterial abundances but these environments are not synchronized across hosts, this would lead to shared abundance correlations over time, but individualized microbiome compositions at any single point in time. Second, the effects of horizontal gene transfer and gene by environment interactions on microbial phenotypes may not be strong enough to substantially alter pairwise microbial associations in the gut. This may be especially true for our main unit of analysis, bacterial ASVs. Because ASVs encompass multiple species and strains, each with somewhat different functional capacities, their dynamics may be buffered against idiosyncrasies driven by horizontal gene transfer and functional redundancy, which affect single strains more strongly than whole species or genera. We would strain-level correlation patterns to be more individualized than those between ASVs. Third, personalized gut microbial compositions may emerge from at least two other phenomena: personalized assembly processes and interactions driven by rare, host-specific strains (which were necessarily excluded from our analyses) (Costello et al., 2012; Walter and Ley, 2011). In general, a logical next step would be to confirm the microbial correlation patterns we observed using culture-based approaches, which will help reveal (in vitro) whether they can be attributed to direct effects of one microbe on another’s growth.

The observation that bacterial correlation patterns are largely shared across hosts was also apparent in one human data set, despite between-study differences in study design, host age, and time scale. Specifically, both the Amboseli baboons and the DIABIMMUNE infant/toddler cohort from Russia (Vatanen et al., 2016) exhibit comparable levels of universality of correlation patterns. This outcome surprised us: because the baboons all live in the same environment and are presumably colonized by similar bacterial strains from that environment, we expected that ecological selection and shared strain functionality should lead to stronger universality in bacteria correlation patterns compared to human infants sampled from different households and who were probably colonized by different strains. We also found that the most universal correlations between bacterial families in baboons tended to be highly universal in human infants/toddlers. Hence, some bacterial families may exhibit consistent microbial relationships within hosts, across host populations, and across host species. Finally, a recent, independent study also identified consistent bacterial correlation patterns across four different populations of human hosts (Loftus et al., 2021). While this study lacked resolution at the level of individual hosts, it did identify a conserved network of positively associated and closely related microbes similar to those we identify in Figure 3. The authors speculate that these conserved associations may indicate strong partner fidelity or obligate partnerships.

We did, however, fail to detect universality in a second human data set reported in Johnson et al., 2019, in which subjects were sampled daily, rather than weekly or monthly. The lack of universality in Johnson et al., 2019, may be due to this difference in sampling time scale, especially if daily abundances and correlations are noisier than covariances modeled over the longer time scales in our study. In support, many fewer of the microbial correlations were stronger than random chance in Johnson et al. as compared to the baboons or children in the DIABIMMUNE cohort. However, without the ability to subsample Johnson et al., 2019, to monthly scales (this data set is only 17 days long), it is impossible to test this prediction. The subjects in Johnson et al., 2019, also consumed substantially different diets from each other, perhaps more so than the children in the DIABIMMUNE cohort, and this inter-host difference in diet may reduce the universality of microbial correlations.

In sum, our study indicates that microbiome personalization may not extend to microbiome community ecology. However, more work is needed to understand how relationships between microbiome taxa are explained by shared internal and external environments, direct and indirect ecological interactions, taxonomic levels (e.g. strains to phyla) and time scales (days to months and years). Future studies should also consider how pairwise bacterial interactions scale up to affect the emergent properties of the community (Levine et al., 2017; Letten and Stouffer, 2019; Friedman et al., 2017). We hope that our longitudinal data set and the new methods we developed as part of this study (e.g. the model of log-ratio dynamics, the assessment of covariation from time-ordered abundance trajectories, and the universality score) will be useful in this enterprise.

## Methods

### Study population and microbiome profiles

The baboon hosts in this study were members of the Amboseli baboon population, which has been studied by the Amboseli Baboon Research Project since 1971 (Alberts and Altmann, 2012). The microbiome compositional profiles are derived from PCR amplification of an ~390-bp-long fragment that encompassed the V4 region of the 16S rRNA gene using primers 515F-806R. These microbiome profiles were previously analyzed in Björk et al., 2022 and Grieneisen et al., 2021. Our analyses use 5534 of these profiles from 56 especially well-sampled baboons, collected over a 13.3-year span between 2000 and 2013 (Figure 1B). Each baboon host in this data set was sampled at least 75 times (mean number of samples=99; range=75–181 samples; median number of days between samples within hosts=20 days; 25th percentile=7 days, 75th percentile=49 days). DNA was extracted from each sample using the MoBio and QIAGEN PowerSoil kit with a bead-beating step. All samples were sequenced on an Illumina HiSeq 2500, with a median read count of 48,827 reads per sample across all 5534 samples (range=982–459,315 reads per sample). Following recommended statistical practices (Gloor et al., 2017), samples were not rarefied, but counts were agglomerated and transformed to additive log-ratios (ALR). Variation in sampling depth and relative abundance were modeled by the method described in the subsequent section. Further details of sample collection, DNA extraction, and sequencing can be found in (Björk et al., 2022; Grieneisen et al., 2021).

### Modeling log-ratio dynamics

Data sets of per-sample taxonomic counts were produced at each of three taxonomic levels, from finest to coarsest: ASV, taxonomic assignments finer than phyla, but above the genus level (e.g. class, order, family), and phylum. At the intermediate and coarsest levels, taxa were agglomerated using phyloseq’s tax_glom() function (McMurdie and Holmes, 2013) such that all sequence variants sharing taxonomic identity at that level were collapsed into a single taxon (e.g. family Bifidobacteraceae). To reduce sparsity in the data set, remove 16S sequences that could represent gene duplications, and focus only on taxa that were prevalent in all 56 hosts, we further filtered as follows: (1) in each of the three taxonomically defined data sets (i.e. ASV, taxa assigned to family/order/class, and phylum), we identified taxa present (i.e. having non-zero abundance) in at least 20% of samples from each host; (2) if a given ASV was >99% genetically similar to another ASV we removed the least abundant of the pair to minimize the risk of including duplicate 16S rRNA gene copies from the same taxa (Vetrovský and Baldrian, 2013); and (3) counts associated with all other taxa were combined into a dummy category, hereafter referred to as ‘other.’ The ‘other’ category therefore includes a combination of rare and host-specific gut microbes. This category was retained in the data set (although not analyzed directly) because ‘other’ counts still inform the precision of the observed relative abundances in our model. See the sub-section titled ‘Filtering out taxon pairs with frequent joint absence’ below for further filtering that was required to avoid biases in estimating correlations between taxon pairs. Characteristics of the filtered data at each taxonomic level are provided in Supplementary file 1a-1c. At the ASV level, these filtering steps eliminate a majority of ASVs and ASV pairs from consideration: from 22,097 unique ASVs before filtering to 107 after filtering. This filtering also retained 12 phyla and 35 taxa at the class/order/family level (Supplementary file 1a-1c).

Our modeling approach is similar to several published methods for modeling microbial time series data. There are three key features from our perspective: the use of log-ratios (discussed above), the use of a state space model, and the Gaussian process component. State space models are useful for modeling a dynamic process that is observed only after the introduction of some measurement (e.g. Joseph et al., 2020). The Gaussian process component helps contend with irregularity in the sampling of our data. Rather than evolving in discrete jumps from one time point to the next, it allowed us to model the change in microbial log-ratio abundances as smoothly flowing through interruptions in observation. Other authors have made similar choices (Äijö et al., 2018). Specifically, estimates of taxon-taxon covariance were obtained from the *basset* model of the ‘fido’ package in R (Silverman et al., 2022). Data for each host took the form of a D×N count matrix, where D gives the number of taxa and N the number of samples collected for a given host. The following model was fit to each host’s count matrix (Y), where $Y_{i}$ represents the counts associated with a single sample:

$$Y_{i}∼\text{Multinomial}\left(\pi_{i}\right)$$

$$\pi_{i}={\text{ALR}}^{-1}\left(\eta_{i}\right)$$

η∼Normal(Λ,Σ,I)

Λ∼GP(Θ[X],Σ,Γ[X])

Σ∼inv-Wishart(Ξ,ν)

The observed relative abundances are considered to be drawn from a multinomial distribution parameterized by a set of proportions (π) which have an analogous representation in the ALR. The dynamics of these log-ratio abundances (η) are described by what amounts to a state space model in the third and fourth lines of the specification above, where a Gaussian process models the evolution of a ‘latent’ state. The matrix Σ captures covariation in log-ratio abundances (the D rows of the observed count matrix). Sample-sample covariation arising from nearness in time (autocorrelation) is modeled by the kernel matrix Γ. Both the kernel matrix and the expected baseline log-ratio abundances (Θ) are parameterized by a set of time-varying covariates X which included the day of sampling (where the date of first sample is defined as zero) and the first three principal components of diet composition, calculated following Björk et al., 2022; Grieneisen et al., 2021, as the diet all juveniles and females living in the host’s social group in the 30 days prior to sample collection. All group members consume highly similar diets as they travel together across the habitat, encountering the same resources at the same time (Björk et al., 2022; Grieneisen et al., 2021). These data are collected via random-order behavioral observations collected two to four times per week on adult females and juveniles in each social group.

The kernel matrix Γ was composed of two component squared exponential kernels. The first, intended to manage sample-sample autocorrelation, was selected to have a bandwidth such that this autocorrelation decayed to a minimum at 90 days. This mirrored the behavior of estimates of sample-sample autocorrelation in the raw data. The second component kernel modeled sample-sample covariance driven by similarity in composition of diet. The relative weight of these effects—autocorrelation and diet—on sample-sample variation was set at 3:1. We fit four alternative versions of our models in order to test the sensitivity of these parameter settings, varying the bandwidth of the squared exponential kernel in such a way as to give minimum sample-sample autocorrelation at either 30 or 90 days. We also varied the proportion of sample-sample covariance driven by diet from 0% to 25% to 50%, and we varied the log scale of total sample-sample variance between 1 and 2. In all cases, estimates of correlation between CLR ASVs were similar, with minimum and maximum *r*^2^ of estimates between ‘canonical’ and alternatively parameterized models of 0.993 and 0.996, respectively. This suggests our findings are reasonably robust to a range of hyperparameter settings.

Posterior inference on this model is performed as described in Silverman et al., 2022, and yields estimates of the distributions of parameters necessary to reconstruct trajectories for all log-ratio taxa across sampling time. In particular, we extract the posterior estimates of one such parameter, Σ, the covariance of ALR taxa, from the fitted models for each host. We convert these covariance matrices over ALR taxa to the CLR form (a simple linear transformation of the matrix). We then normalize estimated CLR covariance matrices to Pearson’s correlation matrices in R using the built-in cov2cor() function.

### Filtering out taxon pairs with frequent joint absence

The ASV-level relative abundance data were sparse, even after filtering low abundance taxa (i.e. those present in <20% of samples in each host). Zeros comprised 29.7% of the ASV-level count matrix, 16.1% of the class/order/family count matrix, and 9.8% of the phylum level count matrix. This abundance of ‘missing’ observations at the ASV level led to a bias in estimates of taxon-taxon correlation: a high-frequency *joint* zero abundances for a pair of taxa increased the apparent positive correlation of those taxa (Figure 1—figure supplement 3). This is because abundances below some minimum level of sensitivity in sampling will be ‘flattened’ to zero, reducing the observed variation for those pairs, over those samples, to zero. This loss of variation leads to a tendency to overestimate the (positive) correlation of these pairs. This trend was observed for our *basset* model (Silverman et al., 2022) when estimating either Pearson’s correlation or proportionality (Quinn et al., 2017). It was also observed in ASV-ASV CLR correlations output by COAT (Cao et al., 2019), which estimates CLR correlation through a sparsity-inducing procedure intended to yield more conservative estimates, and by SparCC (Friedman and Alm, 2012, see Figure 1—figure supplement 3).

To avoid this bias, we restricted our analyses to taxon pairs with strictly less than a 5% frequency of joint absence (i.e. joint zero abundance observations in less than 5% of all samples across hosts) and less than a 50% frequency of absence in either taxon individually across all samples. We illustrate these filtering criteria with a cartoon example involving four taxa, jointly sampled 20 times, indicating presence as an ‘X’ and absence (zero abundance) with a dash:

Taxon A (70% present): X---XXXXXX-XXX-X-XXX

Taxon B (20% present): X---------X--X----X-

Taxon C (50% present): -XXX-----XX-XXXX--X-

Taxon D (65% present): XX-----XX-XX-XX-XXXX

In this example, taxon B would be excluded from analyses by the requirement that taxa be ‘present’ or non-zero in at least half of all samples. Its associations with other taxa (A x B, B x C, or B x D) would be omitted from analyses, leaving pairs A x C, A x D, and C x D. Of these, the rate of joint absence is 5%, 10%, and 15%, respectively, meaning only A x C would pass the filtering criterion of no more than 5% joint absence.

After filtering, 1878 of the original 7750 ASV-ASV pairs remained in this ‘high-confidence’ set. It is this set that we present in all figures and results. This procedure was also carried out at the phylum and class/order/family levels. However, as the frequency of absence was generally small at these higher taxonomic levels, a higher proportion of possible taxon-taxon pairs were included in the high-confidence set: 86.4% (57 of 66 pairs) at the phylum level and 71.0% (473 of 666 pairs) at the class/order/family level.

### Calculating universality scores for taxon-taxon pairs

We devised a universality score for each pair of taxa intended to capture the strength and consistency of taxon-taxon correlations across hosts (Figure 2—figure supplement 4). The majority direction is negative otherwise. This score identifies the sign of the taxon-taxon correlation (positive or negative) that is most common across the 56 hosts (i.e. occurs in >50% of the 56 hosts in the data set). The direction of this sign is the ‘majority correlation sign.’

For a pair of taxa i, let $n_{i}^{\text{maj}}$ be the number of hosts with CLR correlation over pair i with the majority correlation sign for that pair and let n be the total number of hosts. Let R be the subset of estimated CLR correlations for pair i across hosts with the majority sign. The universality score $u_{i}$ for that taxon-taxon pair is then given by

$$u_{i}=\frac{n_{i}^{\text{maj}}}{n}\times \text{median}\left(R\right)$$

This score is the product of the median CLR correlation across hosts and the proportion of hosts with the majority correlation sign, and is bounded between 0 and 1. Scores near 1 indicate strong universality and near-zero scores indicate weak universality. Strong universality can only be achieved by taxon-taxon correlations that are both large in magnitude and highly concordant across hosts.

### Defining a cutoff for significant bacterial correlations and universality scores

We identified correlations stronger than expected by chance using permutations of the data set to define empirical null distributions (Figure 2—figure supplement 3A). Specifically, we permuted the microbial count tables by randomly shuffling taxon identity within each sample 10 times for each of the 56 hosts. This procedure maintained relative abundance patterns within a sample but scrambled the covariance patterns of relative abundances. These randomly generated correlations were pooled into a single reference distribution. The distributions of ASV-level CLR correlations in the original and permuted data are shown in Figure 2—figure supplement 3A. We identified ‘significant’ correlations as those below FDR ≤0.05 (Benjamini-Hochberg), testing against the permuted data.

We applied an analogous permutation test to derive a null distribution for taxon-taxon universality scores. In a single iteration of this permutation procedure, rows and columns of the observed taxon-taxon correlation matrix for each host were shuffled, maintaining the distribution over observed correlations at the host level but randomizing the identity of taxon pairs across hosts. This procedure was repeated 100 times and universality scores were calculated from each of these shuffled data sets to give a single pseudo-null distribution of universality scores. The observed and null distributions of universality scores at the ASV level are shown in Figure 2—figure supplement 3B. We used this empirical null distribution to identify universality scores significantly greater than expected (FDR≤0.05).

### Estimating the ratio of population-level to host-level contributions to observed taxon-taxon correlation patterns

We used simulations to estimate the degree of shared ‘signal’ between hosts in terms of taxon-taxon correlations. Each host’s ‘observed correlations’ were defined as the *basset* estimated maximum a posteriori estimates of CLR ASV correlations for that host. We computed the *mean* correlations across the population using the function estcov() from the shapes package in R (Chen, 2020) and estimated a host-specific contribution to the observed correlations as the residual *difference* between per-host observed and these mean correlations. That is,

observed host correlations=mean population correlations+host residual

For each host, we simulated a hypothetical set of composite taxon-taxon correlations as a convex combination of mean and host residual:

$$\text{composite correlations}=\left(1-\alpha \right)\times \text{mean population correlations}+\alpha \times \text{host residual}$$

A cartoon example of this procedure is given in Figure 3—figure supplement 2. For example, one such simulated set of taxon-taxon correlations might constitute a mixture of 90% host contribution and 10% shared population-level ‘signal’ (*α*=0.9). Alternatively, a small host-level contribution might have *α*=0.1.

For each host, we iterated over increasing proportions of host-level contribution (from 0% to 100%), generating simulated composite correlation matrices according to the formula above. We compared these simulated patterns to those observed for the same host, reasoning that simulated correlation matrices that minimize the distance between the observed correlation matrices and the simulated mixtures provide the best description of the underlying true mixture.

### De-trending for season

Seasonally de-trended data was obtained in the following way. The observed ASV count matrices were CLR-transformed and linear autoregressive models were fit to each CLR-transformed ASV’s series. In these models, wet-dry season oscillation was modeled as a sine wave with a period of 365 days. The magnitude of this component was estimated during model fitting after an offset (in weeks) was estimated in a first step, in order to best align the oscillating seasonal component with the data. Per-ASV models were fit using the following syntax:

$$arima(x=x,xreg=model.matrix(∼factor(host)+sin_{f}(offset,days))[,-1],order=c(1,0,0))$$

where x gives CLR counts for that ASV, the ‘order’ argument of arima enables a single autoregressive component, and the ‘xreg’ argument specifies a covariance matrix. That covariance matrix contains a per-host label giving host-specific offsets for log-ratio abundance and an oscillating seasonal trend through ‘sin_f,’ a function that samples values corresponding to day indices (through ‘days’) from a sine wave with weekly offset (‘offset’) and a period of 365 days. The residuals from these per-ASV model fits were extracted and used as the seasonally ‘de-trended’ data (see Figure 4—figure supplement 1). Correlations across CLR ASV-ASV pairs were estimated from these residual series with the cov() function in R.

### Estimating synchrony

‘Synchrony’ was estimated by sampling aligned microbiome compositional profiles across hosts. We identified all samples collected from pairs of hosts within 1 calendar day. For instance, a sample collected from host F01 on March 14, 2011, could pair with a sample from M04 on March 15, 2011. For all possible pairs of hosts, we selected one such aligned pair of samples, yielding 1540 joint observations of gut microbiome composition. For each such paired sample, one host was arbitrarily designated as host A and the other as host B. The ‘synchrony’ of a given taxon was estimated as the correlation of a taxon’s model-inferred log-ratio abundance across the set of samples from hosts labeled A and the set of samples from hosts labeled B. The cartoon in Figure 4—figure supplement 3 illustrates this sample pairing.

### Enrichment analyses

We performed enrichment analyses for bacterial families and family pairs in several settings. In each case we computed the frequency of ASVs belonging to a given family, or of pairs belonging to a family pair, on a subset of the data. These were compared to the overall frequencies of ASVs belonging to those families or pairs.

To determine the enrichment of families and family pairs in the most universal ASV pairs (Figure 3E; Supplementary file 1e), we calculated the frequencies of ASV families and pairs in the top 5% of pairs by universality scores. Significant enrichment of families or pairs was identified using a one-sided Fisher’s exact test. Multiple test correction was applied as a Benjamini-Hochberg adjustment to observed p-values.

Phylogenetic distances between ASV sequences were calculated with the dist.ml function in the ‘phangorn’ package in R (Schliep, 2011) using default settings for amino acid substitution rates. In Supplementary file 1f, low phylogenetic distance/high median correlation strength pairs were identified as those with phylogenetic distances of less than 0.2 and median correlation strengths of greater than 0.5. Again, significance of these was evaluated against overall frequencies of the same families and pairs.

### Evaluating explanatory factors

#### Variation in taxon-taxon correlation patterns explained by kinship and baseline composition

To evaluate a possible explanatory effect of distances in terms of kinship or baseline gut bacterial composition on distances in terms of taxon-taxon correlation patterns, we applied Mantel tests. However, because population structure can lead to anticonservative p-values (Guillot et al., 2013), we also developed a second simulation-based procedure for evaluating the significance of baseline composition, using a permutation procedure of our own design. First, baseline composition for each host was estimated by transforming all of a given host’s samples to the CLR representation after adding a small fraction (0.5) to remove zeros. The vector of per-taxon averages of these CLR values was used as that host’s ‘baseline’ CLR composition. The Euclidean distances between hosts in terms of these per-host baselines were compared against distances in terms of correlation patterns to give an *r*^2^ value.

In the case of the customized permutation test, this observed result was evaluated against a pseudo-null distribution computed in the following way. The identity of each taxon in the baseline composition was shuffled for each host independently. Euclidean distances across these shuffled baselines were computed and an *r*^2^ value calculated for these distances against the observed distances computed from taxon-taxon correlation patterns. This procedure was repeated 1000 times to give a distribution of ‘random’ *r*^2^ values we used as an empirical null.

#### Variation in taxon-taxon correlation patterns explained by sex and social group

To test whether host sex or social group membership predicted similarity in terms of correlation patterns, we used an ANOVA-like strategy. We calculated the F-statistic, a ratio of between- to within-group variation, on the observed correlation patterns (strictly, the vectorized CLR taxon-taxon correlation matrices; Z in the equation below) and segmented samples into groups defined by either sex or social group. The *F*-statistic was calculated as

$$F=\frac{\text{between-group variation}}{\text{within-group variation}}=\frac{\sum_{i=1}^{K}n_{i}{\left(\bar{Z_{i}}-\bar{Z}\right)}^{2}/K-1}{\sum_{i=1}^{K}\sum_{j=1}^{n_{i}}{\left(Z_{ij}-\bar{Z_{i}}\right)}^{2}/\left(N-K\right)}$$

and significance was evaluated via an *F*-distribution parameterized by the appropriate degrees of freedom. Here, K represents the number of groups (e.g. two, in the case of sex) and N, the total number of hosts. The matrix $\overset{-}{Z_{i}}$ consists of the mean taxon-taxon correlations for group i and $\overset{-}{Z}$, the population mean correlations.

### Comparison to microbiome time series from human populations

We compared our findings to those generated from two human data sets: the DIABIMMUNE project’s infant/toddler cohort from Russian Karelia (Vatanen et al., 2016) and the adult diet-microbiome association study of Johnson et al., 2019. In both cases, count tables were obtained from the project’s public website and subject identity and sampling schedules were available in the associated metadata. We compared each host cohort’s universality at the family/order/class level because this taxonomic level offered the greatest comparative power (10.1% of families/orders/classes overlap between the cohorts compared to just 3.1% of genera and no ASVs). The *basset* model from the ‘fido’ R package (Cullen et al., 2020) was fit to each subject’s data set using model settings analogous to those employed on the Amboseli baboon series: first, only taxa with non-zero counts in at least 20% of all subjects’ series were retained; second, Gaussian process kernel bandwidth settings were chosen in such a way as to encode an expectation of minimum autocorrelation between samples at a distance in time of 90 days. We extracted CLR estimates of taxa at the family level in the same manner as described previously for the Amboseli data set.

## Data Availability

16S rRNA gene sequences are available on EBI-ENA (project 590 ERP119849) and Qiita (study 12949). Analyzed data and code are available on GitHub at: https://github.com/kimberlyroche/rulesoflife (copy archived at Roche, 2023). The following datasets were generated: University of California San Diego Microbiome Initiative 202116S rRNA gene sequencing data from baboon gut microbiomes collected between 2000 and 2014.European Nucleotide ArchiveERP119849 GrieneisenL DasariM GouldTJ BjörkJR GrenierJ YotovaV JansenD GottelN GordonJB LearnNH GesquiereLR WangoTL MututuaRS WarutereJK SiodiL GilbertJA BarreiroLB AlbertsSC TungJ ArchieEA BlekhmanR 2021Gut microbiome heritability is nearly universal but environmentally contingentQiita1294910.1126/science.aba5483PMC837776434244407 The following previously published datasets were used: VatanenT KosticA d'HennezelE 2016DIABIMMUNE three country cohortNCBI BioProjectPRJNA290380 JohnsonAJ 2019Johnson et al. dietary cohortEuropean Nucleotide ArchivePRJEB29065

## Additional files

Supplementary file 1. Supplementary tables.

MDAR checklist
