# Supplementary material for: Universal gut microbial relationships in the gut microbiome of wild baboons
Source: eLife. 2023 May 9;12:e83152. doi: 10.7554/eLife.83152 (PMC10292843; doi:10.7554/eLife.83152)
Supplement: MDAR checklist [file elife-83152-mdarchecklist1.pdf]

eLife
Westbrook Centre
Milton Road
Cambridge CB4 1YG, UK
P 01223 855340
W elifesciences.org
T @elife

# Materials Design Analysis Reporting (MDAR) Checklist for Authors

The MDAR framework establishes a minimum set of requirements in transparent reporting mainly applicable to studies in the life sciences.

*eLife* asks authors to **provide detailed information within their article** to facilitate the interpretation and replication of their work. Authors can also upload supporting materials to comply with relevant reporting guidelines for health-related research (see EQUATOR Network), life science research (see the BioSharing Information Resource), or animal research (see the ARRIVE Guidelines and the STRANGE Framework; for details, see *eLife*'s Journal Policies). Where applicable, authors should refer to any relevant reporting standards materials in this form.

For all that apply, please note **where in the article** the information is provided. Please note that we also collect information about data availability and ethics in the submission form.

## Materials:

| Newly created materials | Indicate where provided: section/figure legend | N/A |
|---|---|---|
| The manuscript includes a dedicated "materials availability statement" providing transparent disclosure about availability of newly created materials including details on how materials can be accessed and describing any restrictions on access. | | N/A |

| Antibodies | Indicate where provided: section/figure legend | N/A |
|---|---|---|
| For commercial reagents, provide supplier name, catalogue number and RRID, if available. | | N/A |

| DNA and RNA sequences | Indicate where provided: section/figure legend | N/A |
|---|---|---|
| Short novel DNA or RNA including primers, probes: Sequences should be included or deposited in a public repository. | The 16S rRNA gene sequences are available on EBI-ENA (project 590 ERP119849) and Qiita (study 12949). This is reported in the data availability section at the end of the main text. | |

| Cell materials | Indicate where provided: section/figure legend | N/A |
|---|---|---|

| | | |
|---|---|---|
| Cell lines: Provide species information, strain. Provide accession number in repository OR supplier name, catalog number, clone number, OR RRID. | | N/A |
| Primary cultures: Provide species, strain, sex of origin, genetic modification status. | | N/A |

| **Experimental animals** | **Indicate where provided: section/figure legend** | **N/A** |
|---|---|---|
| Laboratory animals or Model organisms: Provide species, strain, sex, age, genetic modification status. Provide accession number in repository OR supplier name, catalog number, clone number, OR RRID. | | N/A |
| Animal observed in or captured from the field: Provide species, sex, and age where possible. | In the 6th paragraph of the introduction, we report that the sample size consisted of 5,534 gut microbiome samples from 56 baboons (38 females and 18 males), with 75 to 181 samples per baboon across 6 to 13.3 years, between 2000 and 2013). The baboon hosts were the subject of long-term research on individually recognized animals by the Amboseli Baboon Research Project in Kenya, which has been studying baboon ecology and behavior in the Amboseli ecosystem since 1971.<br><br>In the Materials and Methods, we add that the mean sampling age of subjects ranged from 4.5 to 17.8 years across all 5,534 samples (minimum age ranged from 1.0 to 14.2 years; maximum sampling age ranged from 8.1 to 25.1 years). We also clarify that the host population "is primarily composed of yellow baboons (*Papio cynocephalus*) with some admixture from nearby anubis baboon (*Papio anubis*; also known as the olive baboon) populations." Our sampling time series are shown in Figure 1B and 1C and described in the legend of Figure 1. | |

| Plants and microbes | Indicate where provided: section/figure legend | N/A |
|---|---|---|
| Plants: provide species and strain, ecotype and cultivar where relevant, unique accession number if available, and source (including location for collected wild specimens). | | N/A |
| Microbes: provide species and strain, unique accession number if available, and source. | | N/A |

| Human research participants | Indicate where provided: section/figure legend) or state if these demographics were not collected | N/A |
|---|---|---|
| If collected and within the bounds of privacy constraints report on age, sex, gender and ethnicity for all study participants. | | N/A |

## Design:

| Study protocol | Indicate where provided: section/figure legend | N/A |
|---|---|---|
| If the study protocol has been pre-registered, provide DOI. For clinical trials, provide the trial registration number OR cite DOI. | | N/A |

| Laboratory protocol | Indicate where provided: section/figure legend | N/A |
|---|---|---|
| Provide DOI OR other citation details if detailed step-by-step protocols are available. | The lab protocols rely on commercial kits. In the section on "Study population and generating microbiome profiles", we state that DNA was extracted from each sample using the MoBio and QIAGEN PowerSoil kit following manufacturer instructions, including a bead-beating step. These microbiome profiles were previously published in two papers: Grieneisen et al. 2021 and Björk et al. 2022, which are cited in this section and included in the references. | |

| **Experimental study design (statistics details) *** | | |
|---|---|---|
| **For in vivo studies: State whether and how the following have been done** | **Indicate where provided: section/figure legend. If it could have been done, but was not, write "not done"** | **N/A** |
| Sample size determination | | N/A |
| Randomisation | | N/A |
| Blinding | | N/A |
| Inclusion/exclusion criteria | | N/A |

| **Sample definition and in-laboratory replication** | **Indicate where provided: section/figure legend** | **N/A** |
|---|---|---|
| State number of times the experiment was replicated in the laboratory. | | N/A |
| Define whether data describe technical or biological replicates. | | N/A |

| **Ethics** | **Indicate where provided: section/submission form** | **N/A** |
|---|---|---|
| Studies involving human participants: State details of authority granting ethics approval (IRB or equivalent committee(s), provide reference number for approval. | | N/A |
| Studies involving experimental animals: State details of authority granting ethics approval (IRB or equivalent committee(s), provide reference number for approval. | | N/A |
| Studies involving specimen and field samples: State if relevant permits obtained, provide details of authority approving study; if none were required, explain why. | In the section on "Study population and generating microbiome profiles", we state that permits to collect and export the microbiome samples were obtained from the Kenya Wildlife Service (KWS), the Wildlife Research and Training Institute (WRTI), Kenya's National Commission for Science, Technology and Innovation (NACOSTI), the Convention on International Trade in Endangered Species (CITES), and the US Center for | |

| | | |
|---|---|---|
| | Disease Control and Prevention (US CDC). All work was approved by the Institutional Animal Care and Use Committees (IACUC) at the University of Notre Dame and Duke University. | |

| **Dual Use Research of Concern (DURC)** | **Indicate where provided: section/submission form** | **N/A** |
|---|---|---|
| If study is subject to dual use research of concern regulations, state the authority granting approval and reference number for the regulatory approval. | | N/A |

## Analysis:

| **Attrition** | **Indicate where provided: section/figure legend** | **N/A** |
|---|---|---|
| Describe whether exclusion criteria were pre-established. Report if sample or data points were omitted from analysis. If yes, report if this was due to attrition or intentional exclusion and provide justification. | | N/A |

| **Statistics** | **Indicate where provided: section/figure legend** | **N/A** |
|---|---|---|
| Describe statistical tests used and justify choice of tests. | Our statistical approach leverages a multinomial-logistic normal modeling approach, implemented in the R package 'fido', to infer centered log-ratio (CLR) abundance trajectories for each taxa in each host. We used these trajectories to infer covariances between each pair of taxa in all baboons (represented by covariance matrices). We then converted these covariances to Pearson's correlations and compared bacterial correlation patterns across all hosts, shown as heat maps (red cells are positively correlated taxa; blue cells reflect negatively correlated taxa). These correlations are analyzed using several statistical approaches. Our overall approach is summarized in Figure 1A, and all statistics are described in detail in the | |

| | | |
|---|---|---|
| | Materials and Methods, especially the sections titled, "Modeling log-ratio dynamics", "Calculating universality scores for taxon-taxon pairs", "Defining a cutoff for significant bacterial correlations and universality scores", "Estimating the ratio of population-level to host-level contributions to observed taxon-taxon correlation patterns", "Estimating synchrony", "Enrichment analyses", "Evaluating explanatory factors", and "Comparison to microbiome time series from human populations". | |

| **Data availability** | **Indicate where provided: section/submission form** | **N/A** |
|---|---|---|
| For newly created and reused datasets, the manuscript includes a data availability statement that provides details for access (or notes restrictions on access). | All data and code are publicly available. 16S rRNA gene sequences for the Amboseli baboons are available on EBI-ENA (project 590 ERP119849) and Qiita (study 12949). Analyzed data and code are available on GitHub at: https://github.com/kimberlyroche/rulesoflife<br><br>We also re-analyze data from Vatanen et al. (2016), available at NCBI BioProject ID PRJNA290380 and data from Johnson et al. (2019) available at ENA BioProject ID PRJEB29065. | |
| When newly created datasets are publicly available, provide accession number in repository OR DOI and licensing details where available. | | N/A (these data are not newly created) |
| If reused data is publicly available provide accession number in repository OR DOI, OR URL, OR citation. | 16S rRNA gene sequences are available on EBI-ENA (project 590 ERP119849) and Qiita (study 12949). Analyzed data and code are available on GitHub at: https://github.com/kimberlyroche/rulesoflife<br><br>We also re-analyze data from | |

| | | |
|---|---|---|
| | Vatanen et al. (2016), available at NCBI BioProject ID PRJNA290380 and data from Johnson et al. (2019) available at ENA BioProject ID PRJEB29065. | |

| **Code availability** | **Indicate where provided: section/figure legend** | **N/A** |
|---|---|---|
| For any computer code/software/mathematical algorithms essential for replicating the main findings of the study, whether newly generated or re-used, the manuscript includes a data availability statement that provides details for access or notes restrictions. | Analyzed data and code are available on GitHub at: https://github.com/kimberlyroche/rulesoflife | |
| Where newly generated code is publicly available, provide accession number in repository, OR DOI OR URL and licensing details where available. State any restrictions on code availability or accessibility. | Analyzed data and code are available on GitHub at: https://github.com/kimberlyroche/rulesoflife | |
| If reused code is publicly available provide accession number in repository OR DOI OR URL, OR citation. | Analyzed data and code are available on GitHub at: https://github.com/kimberlyroche/rulesoflife | |

## Reporting:

The MDAR framework recommends adoption of discipline-specific guidelines, established and endorsed through community initiatives.

| **Adherence to community standards** | **Indicate where provided: section/figure legend** | **N/A** |
|---|---|---|
| State if relevant guidelines (e.g., ICMJE, MIBBI, ARRIVE, STRANGE) have been followed, and whether a checklist (e.g., CONSORT, PRISMA, ARRIVE) is provided with the manuscript. | | N/A |

---

* We provide the following guidance regarding transparent reporting and statistics; we also refer authors to Ten common statistical mistakes to watch out for when writing or reviewing a manuscript.

**Sample-size estimation**

- You should state whether an appropriate sample size was computed when the study was being designed
- You should state the statistical method of sample size computation and any required assumptions
- If no explicit power analysis was used, you should describe how you decided what sample (replicate) size (number) to use

**Replicates**

- You should report how often each experiment was performed
- You should include a definition of biological versus technical replication
- The data obtained should be provided and sufficient information should be provided to indicate the number of independent biological and/or technical replicates
- If you encountered any outliers, you should describe how these were handled
- Criteria for exclusion/inclusion of data should be clearly stated
- High-throughput sequence data should be uploaded before submission, with a private link for reviewers provided (these are available from both GEO and ArrayExpress)

**Statistical reporting**

- Statistical analysis methods should be described and justified
- Raw data should be presented in figures whenever informative to do so (typically when N per group is less than 10)
- For each experiment, you should identify the statistical tests used, exact values of N, definitions of center, methods of multiple test correction, and dispersion and precision measures (e.g., mean, median, SD, SEM, confidence intervals; and, for the major substantive results, a measure of effect size (e.g., Pearson's r, Cohen's d)
- Report exact p-values wherever possible alongside the summary statistics and 95% confidence intervals. These should be reported for all key questions and not only when the p-value is less than 0.05.

**Group allocation**

- Indicate how samples were allocated into experimental groups (in the case of clinical studies, please specify allocation to treatment method); if randomization was used, please also state if restricted randomization was applied
- Indicate if masking was used during group allocation, data collection and/or data analysis
