# Supplementary material for: Universal gut microbial relationships in the gut microbiome of wild baboons
Source: eLife. 2023 May 9;12:e83152. doi: 10.7554/eLife.83152 (PMC10292843; doi:10.7554/eLife.83152)
Supplement: Peer review history

# Editor report: Editor's evaluation

**DOI:** 10.7554/eLife.83152.sa0

**Authors:** Dario Riccardo Valenzano

**Affiliations:** https://ror.org/039a53269 Leibniz Institute on Aging Germany

In this work, Roche et al. study a 13-year long time series of microbiome samples from wild baboons from Kenya. This data allows disentangling ecological dynamics within and across individuals in a way that has never been done before. The authors show that the ecological relationships among baboon gut bacteria, measured through a correlation based on covariation, are largely universal (similar within and across host individuals) and that the most universally covarying taxa are almost always positively associated with each other. This work is foundational in its compelling effort to generate a rigorous method to evaluate co-abundance dynamics in longitudinal microbiome data. The approach taken will likely inspire developments that will sharpen the capacity to extract co-varying microbial features, taking into account seasonality, diet, age, relatedness, and more.

# Decision letter

**DOI:** 10.7554/eLife.83152.sa1

**Affiliations:** https://ror.org/039a53269 Leibniz Institute on Aging Germany; https://ror.org/052gg0110 University of Oxford United Kingdom; https://ror.org/03qxff017 The Hebrew University of Jerusalem Israel

Our editorial process produces two outputs: (i) public reviews designed to be posted alongside the preprint for the benefit of readers; (ii) feedback on the manuscript for the authors, including requests for revisions, shown below. We also include an acceptance summary that explains what the editors found interesting or important about the work.

**Decision letter after peer review:**

Thank you for submitting your article "Universal gut microbial relationships in the gut microbiome of wild baboons" for consideration by *eLife*. Your article has been reviewed by 3 peer reviewers, including Dario Riccardo Valenzano as the Reviewing Editor and Reviewer #1, and the evaluation has been overseen by Wendy Garrett as the Senior Editor. The following individuals involved in the review of your submission have agreed to reveal their identity: Aura Raulo (Reviewer #2); Oren Kolodny (Reviewer #3).

The reviewers have discussed their reviews with one another, and the Reviewing Editor has drafted this to help you prepare a revised submission.

Essential revisions:

1. Is the covariation data zero-inflated?

2. Did the authors find or analyze the age-dependency in microbial dynamics, i.e. whether baboon age is characterized by specific microbial associations that are not equally maintained across all age groups? More extensively: are there taxonomic covariations that are (i) lost or (ii) acquired during aging?

3. The pairwise species correlations can be explained in (at least) two ways: the species have positive relations in some way (e.g. one is providing something necessary for the other), or the two simply like to be in the same kind of habitat. The "same kind of habitat" may refer to both a similar broad environment of the host (including diet, soil type, etc) OR a similar within-host environment, i.e. host physiology, gut pH, immune status, etc. I would suggest having a discussion of these alternative explanations (and perhaps others) early on, and reference to this discussion in later interpretations of findings, throughout the results and Discussion sections.

4. A graphical summary that would explain the consensus model for the temporal dynamics of microbial pair associations would help clarify the take-home message to a broader audience.

*Reviewer #1 (Recommendations for the authors):*

I very much liked this work and I congratulate the authors for their contribution to the field of microbiome ecology.

I would suggest better clarifying the novelty compared to previous analyses performed by the authors on this dataset.

It was not clear to me whether the authors found or analyzed the age-dependency in microbial dynamics, i.e. whether baboon age is characterized by specific microbial associations that are not equally maintained across all age groups. More extensively: are there taxonomic covariations that are (i) lost or (ii) acquired during aging?

A graphical summary that would explain the consensus model for the temporal dynamics of microbial pair associations would help clarify the take-home message to a broader audience.

I would like to kindly ask the authors to explain their chosen criteria for authorship. In particular, the authors should clarify whether the contribution of any of the scientific collaborators in Kenya could be worthy of inclusion in the authors' list. To date, the support that goes into field work by local scientists and trainees is not sufficiently acknowledged by foreign researchers, and a more inclusive and less exploitative authorship system can make a difference in developing countries, promoting long-term scientific excellence.

*Reviewer #2 (Recommendations for the authors):*

• Regarding my worries over the effect of 0-0 links on the positive correlation assessment, if your covariation data is zero-inflated, I suggest you would consider whether a correlation measure based on SparCC-method (See: ), such as SpiecEASI (ref) might be a more robust way of estimating covariation through sparse inverse covariance. If your covariation data is magically not zero-inflated, I would suggest either making it into a bigger thing in the text or considering using the SparCC methods anyway, as they would allow you to have more of the rare taxa in the data. Alternatively, you could just show how much of your positive and negative correlation patterns respectively were influenced by whether or not you consider double zeros or any zeros in the data. You could do this either with separate models or within one zero-inflated hurdle model. If you can show that the pattern prevails even when you only compare non-zero abundances, that would make your correlation method that much more convincing.

• 10 permutations to address the significance of the correlations sounds la quite a low number to me. Would you have the computing power to do 100? I do not really understand how you get to p <0.05 with just 10 permutations.

• You could add a sentence to the abstract to elaborate on why we would expect ecological relationships to be individualized in the first place. I was a bit confused reading the abstract about why is this a matter worth such detailed exploration, but your introduction really convinced me. If you could add something from lines 82-91 into the abstract, it would perhaps make it more intriguing

• You show that population-level signatures contributed almost twice the weight as host-level signatures on correlation patterns. I think this is convincing. But I do think there seems to still be surprisingly much individual variation in ecological associations. I would have expected them to be even more universal, to be honest. I think it would be interesting to add also a discussion on why some taxa are strongly but inconsistently correlated – do these taxa have something special about them? Are they more generalist? Or do they have more positive links (can depend on many others rather than fully dependent on one other taxon)?

• Your universality score takes continuous correlation strength within individual and proportion of hosts with a majority sign as input. I like it, but wonder if you could capture even more of the variation in your data by also using a continuous measure of cross-sectional correlation consistency? Like additive correlation strength in the majority sign relative to additive correlation strength in the non-majority sign. Just a thought though.

• Lines 143-146, you could emphasize that if taxa covariation is driven by selection imposed by the host/environmental, then we would expect phylogenetically or phenotypically similar taxa to be positively covarying. If, on the other hand, covariation patterns were more driven by ecological interactions between taxa, we might expect positive covariation to be not more common in phylogenetically close taxa or less common based on competitive exclusion. Or is there some evidence that phylogenetically close taxa cross-feed more with each other or such?

• Lines 254-255, you write "Note, that the correlation strength for a given pair of ASVs was only weakly predicted by bacterial abundance " – Does this mean it was mostly driven by co-occurrence or that the covariation in abundances was sensitive to overall abundance? I guess the latter. More clarity would be good.

• Line 406, you write " Universality in Amboseli is not solely explained by seasonality or synchrony " – I think this is a bit manipulative title. There is quite a bit of evidence there for seasonality and synchrony and other evidence for environmental of host physiology-related selection driving covariation patterns (such as the fact that positive covariation is more common in phylogenetically close pairs). I feel like someone else could have formulated these results by downplaying the ecological relationships notion and emphasizing the selective effects notion. There is a bit of a tone here like you would prefer the ecological network effect over the environmentally driven covariation. I suggest rewording this to be a bit more neutral, such as "Universality is partially explained by seasonality and synchrony". And also mention that there may be other selective effects (like those related to individual variation in host physiology?) that you did not test but might feed into the selective effects driving covariation.

• Lines 465-467: I am not entirely convinced that the lack of similar patterns in the Johnson data set is likely explained by the different sampling frequencies. Was there much less temporal variation in the Johnson data set? To back up the statement that higher sampling frequency would be the reason the Johnson data set has dissimilar covariation between taxa compared to yours, perhaps you could show that the temporal variation in this data set was different from the baboon one and show that these covariation patterns were sensitive to timescale by subsampling either data to create mock data sets with different sampling frequency and see how this would change the inference of ecological associations. In general, I would tone down the generalizability to humans -conclusions a bit since only one of your data sets showed this, and it is in infants, who have an ecologically more unstable microbiome than adult humans.

• Lines 540-554. Can you clarify why exactly should environmental variation decrease the universality of ecological associations? I would imagine that environmental variation can expand the space of microbial covariation and if universality is driven by covariation due to environmental selection, then this should be maximal when there is broader space for environmental variation to exist. You mentioned in the intro that "genotype by environment interactions, and priority effects-can lead microbiome taxa to fill different ecological roles in different hosts", could you explain a bit more somewhere how this translate to more environmental variation leading to less clear covariation between taxa?

• Lines 575-576 What about individual variation in host physiology?

• Line 633 How much was the sparsity reduced?

• Line 643 Seems very cool but I cannot fully critically evaluate the statistical robustness of this modeling framework

*Reviewer #3 (Recommendations for the authors):*

• Good abstract, presentation, and introduction.

• Figure 2: perhaps mark in panel A what the threshold for significant positive/negative correlations was.

• Positive correlation – as you note in several places – can be explained in (at least) two ways: the species have positive relations in some way (e.g. one is providing something necessary for the other), or the two simply like to be in the same kind of habitat, so when it is good for one it's also good for the other. You are aware of this, as both possibilities are mentioned in several places, but it seems that sometimes you choose to offer one and sometimes the other, with no clear reason (e.g. you propose that correlations at the phylum level are due to environmental preferences – lines 217-219 – but this explanation is in contrast to the strong emphasis on microbe-microbe interactions that is found throughout).

• I would suggest having a discussion of these alternative explanations (and perhaps others) early on, and reference to this discussion in later interpretations of findings, throughout the results and Discussion sections.

(you are clearly aware of this, e.g. in line 407; I suggest discussing this topic in the introduction and referring to it throughout. This would help readers who aren't aware of the extensive research/discussion/debate about these questions in microbial ecology, landscape ecology, and elsewhere).

• A brief mention/clarification (at least) of causality vs. correlation would be a good idea in this context. Even if clear correlations are found between taxa, this doesn't imply causation, of course. Perhaps discuss in future directions the importance of intervention/manipulation studies to test for causation.

• There's quite a large literature in ecology, particularly microbial ecology, that deals with the link between pairwise interactions between bacteria within a larger consortium of species, and whether inferences can be made from pairwise interactions to more complex scenarios; consider referring to some of this literature and perhaps offering a discussion of your results in light of the insights proposed there. Some such studies (I'm not from the field, there may be better ones) are:

https://www.nature.com/articles/nature22898

https://onlinelibrary.wiley.com/doi/full/10.1111/ele.13211

https://www.nature.com/articles/s41559-017-0109

Also, have a look at one or two possibly relevant studies by Andrew Letten.

• A possible interpretation of the finding that correlations, when exist, tend to be positive: if the driver of significant correlations is the environment, and not positive species' interactions, then this observation might be expected: pairs of species that share environmental preferences will be positively correlated, and pairs of species that prefer different environments would be uncorrelated (and not negatively correlated).

In other words: there is only one way in which environmental preferences can be similar, but many ways in which two environmental preferences can differ (and also an environment is similar to itself in all dimensions, but there are many dimensions in which two environments can differ). "All happy families are alike, but every unhappy family is unhappy in its own way (Leo Tolstoy, Anna Karenina, 1878)".

In a sense, this observation should thus perhaps be viewed as support of the hypothesis that the driver of the positive correlations you find is shared environmental preferences and not species-species interactions. I think. Consider.

• 545-555: If true, the positive correlations are due to shared preferences of environment, it perhaps makes sense that the children dataset, in which children differ quite a bit (more than pairs of baboons), shows a strong signal: the fact that children are different should create high diversity in the overall dataset, and when two children happen to be similar in the conditions they create in their guts – this (and the respective positive correlations between pairs of species that like these specific conditions) would stand out particularly significantly above all this noise. Maybe. This requires some deeper thought, so consider. ((this may be analogous to assessing heritability of traits – heritability seems to decrease – sometimes to the point of being non-significant/below detection level – in a homogenic population, and heritability estimates are higher when the population is diverse))

• 572 – 576 (starting with "We surmise that most") – I would be more cautious about this statement.

I tend to think that the driver of the correlation universality in your data is shared environmental preferences, and – apart from the point I made above – I think this is also particularly likely in light of the phylogenetic signal that you found (it makes sense that phylogenetically related species have similar environmental preferences, stemming from homology; this seems to me more parsimonious compared to the possibility that related species tend to be more supportive of one another for some reason, even though I can come up with some handwaving explanations that could support this if I really had to).

The "environment" in question is the one in the gut. Thus, controlling for diet or seasonal drivers is good, but far from ruling out that there are shared environments that are driving the signal; for that, you'd need to control for the extent to which pairs of host individuals tended to have more similar pH, hormonal status, immune activation (and its profile) and so on.

• 589: There seems to be a problem with this sentence. Look at the "the fact that…" – seems like something is missing.

• Methods: I'd elaborate a bit further about the sequencing, e.g. whether you rarefied samples or accounted for uneven read counts in another way, and which 16s regions were amplified (and/or what their length was – amplifying just V3, for example, would lead to a very different ASV resolution from amplifying V3+V4).

# Reply: Author response

**DOI:** 10.7554/eLife.83152.sa2

Essential revisions:

1. Is the covariation data zero-inflated?

We very much appreciate the suggestion to check for zero-inflation and R2’s detailed comments on this topic below. We are embarrassed that we didn’t consider how zero inflation might affect the correlation patterns in our original analyses. Indeed, zero inflation biased our correlations such that taxon pairs with a high frequency of joint zero observations (i.e., where both members of the pair often had very low or zero abundances) tended to be positively correlated (Figure 1 —figure supplement 3, shown below). This is because, as R2 suggests, zero inflation in the data lends more weight to positive links than negative links.

To address this problem in the revised manuscript, we now restrict our analyses to taxon pairs with strictly less than a 5% frequency of joint absence (i.e., joint zero-abundance observations in less than 5% of all samples across hosts, to the left of the dashed line in Figure 1 —figure supplement 3). We further restricted to pairs with less than a 50% frequency of absence in either taxon individually across all samples. We explain the rationale behind our filtering criteria in lines 735-764. After filtering, 1,878 of the original 7,750 ASVASV pairs were retained in our analyses (86.4% of the original pairs at the phylum level; 71.0% at the class/order/family level).

Nearly all of our results are robust to these changes. The only two results that differ from the original submission are (i) consistent with the 2^nd^ reviewer’s suspicions, we no longer see a bias towards positive correlations in our most universal taxa pairs, and (ii) we no longer see enrichment for members of the same bacterial family in the most-universal pairs. However, our other main results are the same: most bacterial correlations are weak and negative; each baboon reflects a mixture of idiosyncratic and shared correlation patterns, but shared patterns dominate by almost 2-fold; host pairs with the most similar bacterial correlation patterns also had similar microbiome taxonomic compositions and tended to be genetic relatives.

2. Did the authors find or analyze the age-dependency in microbial dynamics, i.e. whether baboon age is characterized by specific microbial associations that are not equally maintained across all age groups? More extensively: are there taxonomic covariations that are (i) lost or (ii) acquired during aging?

We agree that the effects of host age on microbial dynamics are an interesting topic. Host age may predict the overall strength of microbial relationships, and prior studies suggest that microbial relationships may become more individualized with age [5, 6]. To test these ideas, we added two new paragraphs to the results (lines 395-415) and three new supplementary figures (Figure 5 —figure supplements 1, 2 and 3). Briefly, we found no evidence that microbial correlations get stronger or weaker with age (Figure 5 —figure supplement 1).

Further, we found no strong differences in the degree of “personalized” correlation patterns across age groups (Figure 5 —figure supplement 2).

3. The pairwise species correlations can be explained in (at least) two ways: the species have positive relations in some way (e.g. one is providing something necessary for the other), or the two simply like to be in the same kind of habitat. The "same kind of habitat" may refer to both a similar broad environment of the host (including diet, soil type, etc) OR a similar within-host environment, i.e. host physiology, gut pH, immune status, etc. I would suggest having a discussion of these alternative explanations (and perhaps others) early on, and reference to this discussion in later interpretations of findings, throughout the results and Discussion sections.

Thank you for this suggestion. We now discuss this topic in the Discussion section (lines 552 to 567 and lines 580 to 583). These edits clarify that our correlations can arise from two non-exclusive processes: ecological interactions between species or correlated responses to environmental gradients. While our approach corrects for some of these factors, namely diet, season, and synchronized dynamics between hosts, we did not account for key environmental gradients within hosts—especially immune profiles, intestinal pH, and hormones. We now discuss how these differences could explain some of our observations, especially the finding that close genetic relatives have similar dynamics and that the most consistent ASV-level correlations are between phylogenetically related taxa. We have also made small updates to clarify these ideas in the Introduction (lines 129 to 130; 139 to 141) and the Results (lines 230 to 233; 351 to 354; text starting at line 430).

4. A graphical summary that would explain the consensus model for the temporal dynamics of microbial pair associations would help clarify the take-home message to a broader audience.

We are not confident we understood what you meant by the “consensus model for temporal dynamics of microbial pair associations”, but we think you mean our universality score. In response, we have added a new Figure 2 —figure supplement 4 which illustrates this score.

Reviewer #1 (Recommendations for the authors):

I very much liked this work and I congratulate the authors for their contribution to the field of microbiome ecology.

Thank you for your supportive comments.

I would suggest better clarifying the novelty compared to previous analyses performed by the authors on this dataset.

We agree.

It was not clear to me whether the authors found or analyzed the age-dependency in microbial dynamics, i.e. whether baboon age is characterized by specific microbial associations that are not equally maintained across all age groups. More extensively: are there taxonomic covariations that are (i) lost or (ii) acquired during aging?

We have added age-dependency in the revised text (lines 395-415). Please see our response in R2 above, as well as new Figure 5 —figure supplements 1, 2, and 3. Briefly, we found no evidence that microbial correlations get stronger or weaker with age (Figure 5 —figure supplement 1) and no strong differences in the degree of “personalized” correlation patterns across age groups (Figure 5 —figure supplement 2). We did, however, find small differences in the strength of some microbial correlations between age (Figure 5 —figure supplement 3).

A graphical summary that would explain the consensus model for the temporal dynamics of microbial pair associations would help clarify the take-home message to a broader audience.

Please see our response above.

I would like to kindly ask the authors to explain their chosen criteria for authorship. In particular, the authors should clarify whether the contribution of any of the scientific collaborators in Kenya could be worthy of inclusion in the authors' list. To date, the support that goes into field work by local scientists and trainees is not sufficiently acknowledged by foreign researchers, and a more inclusive and less exploitative authorship system can make a difference in developing countries, promoting long-term scientific excellence.

This is a good question and one we have discussed much among ourselves. Several of our Kenyan research team (Raphael Mututua, Kinyua Warutere, Long’ida Siodi, and Tim Wango) played an essential role on collecting and processing the fecal samples used to generate the microbiome compositional profiles used in this analysis. Raphael Mututua, Kinyua Warutere, and Long’ida Siodi also contributed to collecting the demographic, behavioral, and ecological covariates we analyzed. We therefore included these four authors on the two original publications arising from this data set (citations below). However, because this current paper is a re-analysis of these previously published data, and because these authors did not contribute to designing or implementing these analyses or to writing or revising the paper, we felt it would be inappropriate to include them on this manuscript.

Citations for the first papers to publish analyses of this data set (references 43 and 44 in the manuscript):

Grieneisen L., Dasari M., Gould T.J., Björk J.R., Grenier J., Yotova V., Jansen D., Gottel N., Gordon J.B., Learn N.H., Gesquiere L.R., Wango T.L., Mututua R.S., Warutere J.K., Siodi L., Gilbert J.A., Barreiro L.B., Alberts S.C., Tung J., Archie E.A., Blekhman R. 2021. Gut microbiome heritability is nearly universal but environmentally contingent. *Science* 373:181-186

Björk J.R., Dasari M., Roche, K., Grieneisen L.,Gould T.J., Grenier J.C., Yotova V., Gottel N., Jansen D., Gesquiere L.R., Gordon J.B., Learn N.H., Wango T.L., Mututua R.S.,

Warutere J.K., Siodi L., Mukherjee, S., Barreiro L.B., Alberts S.C., Gilbert J.A., Tung J., Blekhman R., Archie E.A. 2022. Synchrony and idiosyncrasy in the gut microbiome of wild baboons. *Nature Ecology and Evolution* 6: 955–964

Reviewer #2 (Recommendations for the authors):

• Regarding my worries over the effect of 0-0 links on the positive correlation assessment, if your covariation data is zero-inflated, I suggest you would consider whether a correlation measure based on SparCC-method (See: ), such as SpiecEASI (ref) might be a more robust way of estimating covariation through sparse inverse covariance. If your covariation data is magically not zero-inflated, I would suggest either making it into a bigger thing in the text or considering using the SparCC methods anyway, as they would allow you to have more of the rare taxa in the data. Alternatively, you could just show how much of your positive and negative correlation patterns respectively were influenced by whether or not you consider double zeros or any zeros in the data. You could do this either with separate models or within one zero-inflated hurdle model. If you can show that the pattern prevails even when you only compare non-zero abundances, that would make your correlation method that much more convincing.

Thank you. In response to this comment, we substantially revised our filtering criteria and re-analyzed the data.

• 10 permutations to address the significance of the correlations sounds la quite a low number to me. Would you have the computing power to do 100? I do not really understand how you get to p <0.05 with just 10 permutations.

We have added more detail to the text starting at line 202 to clarify our permutation approach. Specifically, to generate an expectation of the strength of bacterial correlations possible by chance, we used a permutation procedure that randomly shuffled the taxonomic identities within each sample of the bacterial count table 10 times for each of the 56 hosts (560 total permutations). We then estimated correlations for these permuted pairs to generate an empirical null distribution of randomly generated taxon-taxon correlations. Hence, the “significance” of individual taxon-taxon correlations was evaluated against a very large, pooled distribution of randomly generated correlations.

• You could add a sentence to the abstract to elaborate on why we would expect ecological relationships to be individualized in the first place. I was a bit confused reading the abstract about why is this a matter worth such detailed exploration, but your introduction really convinced me. If you could add something from lines 82-91 into the abstract, it would perhaps make it more intriguing

We have added two sentences to the abstract (starting on line 42), which read, “However, whether bacterial relationships are generalizable across hosts or personalized to individual hosts is debated. Several eco-evolutionary processes could personalize microbiome community ecology, but the few studies that have tested this idea find that bacterial interactions are largely consistent (i.e., “universal”) across hosts”

• You show that population-level signatures contributed almost twice the weight as host-level signatures on correlation patterns. I think this is convincing. But I do think there seems to still be surprisingly much individual variation in ecological associations. I would have expected them to be even more universal, to be honest. I think it would be interesting to add also a discussion on why some taxa are strongly but inconsistently correlated – do these taxa have something special about them? Are they more generalist? Or do they have more positive links (can depend on many others rather than fully dependent on one other taxon)?

Actually, we did not find any pairs that were strongly and inconsistently correlated. For instance, in Figures 3A and 3B, the taxa with inconsistent correlation signs (far left on the xaxis) have only weak median correlations within hosts. To clarify this result, we now mention it in the abstract (line 52-54): “taxon pairs that had inconsistent correlation signs (either positive or negative) in different hosts always had weak correlations within hosts.” In addition, we have revised the text starting at line 259 to clarify that we do not observe any pairs of taxa that are strongly and inconsistently correlated. This text reads, “First, in support of the idea that ASVs do not exhibit vastly different correlative relationships in different hosts, no taxon pairs were strongly and *inconsistently* correlated across hosts (Figures 3A and 3B; Figure 3 – —figure supplement 1A). Instead, the ASV pairs that had inconsistent correlation signs across hosts always had weak and often non-significant median absolute correlation coefficients within hosts (Figures 3A and 3B)”.

• Your universality score takes continuous correlation strength within individual and proportion of hosts with a majority sign as input. I like it, but wonder if you could capture even more of the variation in your data by also using a continuous measure of cross-sectional correlation consistency? Like additive correlation strength in the majority sign relative to additive correlation strength in the non-majority sign. Just a thought though.

We think this is an interesting idea, but we were concerned that our initial universality score was already a little challenging for readers to understand. We suspect (but did not confirm) that your revised score might show qualitatively similar patterns to the original score, and in the end, we did not include this suggestion in the revised paper.

• Lines 143-146, you could emphasize that if taxa covariation is driven by selection imposed by the host/environmental, then we would expect phylogenetically or phenotypically similar taxa to be positively covarying. If, on the other hand, covariation patterns were more driven by ecological interactions between taxa, we might expect positive covariation to be not more common in phylogenetically close taxa or less common based on competitive exclusion. Or is there some evidence that phylogenetically close taxa cross-feed more with each other or such?

We have updated this prediction (line 151) to read: “Third, we expected to observe positive correlations between taxa that are close phylogenetic relatives. This is because related bacteria may have similar functional properties and hence similar ecological relationships with other members of the community. They may also have dynamics that are driven by similar selective forces imposed by the host or host’s environment. Alternatively, competitive exclusion may lead closely related taxa to exhibit neutral or negative relationships.”

• Lines 254-255, you write "Note, that the correlation strength for a given pair of ASVs was only weakly predicted by bacterial abundance " – Does this mean it was mostly driven by co-occurrence or that the covariation in abundances was sensitive to overall abundance? I guess the latter. More clarity would be good.

Your interpretation is correct. We meant that covariation is sensitive to overall abundance but the effect is weak. We clarified the text starting at line 271 to read, “Note, that the correlation for a given pair of ASVs was only weakly predicted by bacterial abundance (r=0.129 and r=0.223 for the more and less abundant partner in a pair respectively; p < 0.0001 both). While this effect was statistically significant, it explained only 6% of the variance in median correlation.”

• Line 406, you write " Universality in Amboseli is not solely explained by seasonality or synchrony " – I think this is a bit manipulative title. There is quite a bit of evidence there for seasonality and synchrony and other evidence for environmental of host physiology-related selection driving covariation patterns (such as the fact that positive covariation is more common in phylogenetically close pairs). I feel like someone else could have formulated these results by downplaying the ecological relationships notion and emphasizing the selective effects notion. There is a bit of a tone here like you would prefer the ecological network effect over the environmentally driven covariation. I suggest rewording this to be a bit more neutral, such as "Universality is partially explained by seasonality and synchrony". And also mention that there may be other selective effects (like those related to individual variation in host physiology?) that you did not test but might feed into the selective effects driving covariation.

These comments prompted us to give a more even hand to both explanations for our data. We have revised the sub-title for this section, which now reads, “Universality in Amboseli is not well explained by microbes’ shared responses to diet, season, or synchronized dynamics”. We also clarify in line 430 that “without experiments, we cannot disentangle whether our observed bacterial correlations are due to ecological interactions between bacterial species or to shared responses to environmental gradients, either inside or outside the host. We agree that host environments could play a big role in the patterns we see. This is noted in line 433 and discussed in a new paragraph in the discussion, which starts at line 552. In this section, we state that environmental differences between hosts in the host gut are likely to explain, at least in part, the observations that close genetic relatives have similar dynamics and that the most consistent ASV-level correlations were between phylogenetically related taxa.

• Lines 465-467: I am not entirely convinced that the lack of similar patterns in the Johnson data set is likely explained by the different sampling frequencies. Was there much less temporal variation in the Johnson data set? To back up the statement that higher sampling frequency would be the reason the Johnson data set has dissimilar covariation between taxa compared to yours, perhaps you could show that the temporal variation in this data set was different from the baboon one and show that these covariation patterns were sensitive to timescale by subsampling either data to create mock data sets with different sampling frequency and see how this would change the inference of ecological associations. In general, I would tone down the generalizability to humans -conclusions a bit since only one of your data sets showed this, and it is in infants, who have an ecologically more unstable microbiome than adult humans.

Briefly, in the Discussion in line 619, we now clarify that it is not possible to subsample Johnson et al. [7] to monthly scales because the data set is only 17 days long.

• Lines 540-554. Can you clarify why exactly should environmental variation decrease the universality of ecological associations? I would imagine that environmental variation can expand the space of microbial covariation and if universality is driven by covariation due to environmental selection, then this should be maximal when there is broader space for environmental variation to exist. You mentioned in the intro that "genotype by environment interactions, and priority effects-can lead microbiome taxa to fill different ecological roles in different hosts", could you explain a bit more somewhere how this translate to more environmental variation leading to less clear covariation between taxa?

This paragraph of the discussion has been edited. The text starting at line 599 now states, “This outcome surprised us: because the baboons all live in the same environment and are presumably colonized by similar bacterial strains from that environment, we expected that ecological selection and shared strain functionality should lead to stronger universality in bacteria correlation patterns compared to human infants sampled from different households and who were probably colonized by different strains.”

We have also updated the text in the discussion that mentions the role of genotype by environment interactions and how they might lead to personalized covariation between taxa. The text starting at line 87 states, “For instance, several common community and evolutionary processes—such as horizontal gene transfer and priority effects—can lead microbiome taxa to fill different ecological roles in different hosts [8-13]. Further, genotype by environment interactions and plasticity could lead some microbes to adopt contextdependent metabolisms and ecological roles depending on their microbial neighbors or other aspects of the environment [14-17].”

• Lines 575-576 What about individual variation in host physiology?

This sentence is no longer in the paper.

• Line 633 How much was the sparsity reduced?

Please see our response to Essential revision 1 above

• Line 643 Seems very cool but I cannot fully critically evaluate the statistical robustness of this modeling framework

We have added a few lines starting at line 677 to place our approach in context. Essentially, our model resembles several published methods for modeling microbial time series data. There are three key features from our perspective: the use of log-ratios, the use of a state space model, and the Gaussian process component. Log-ratios have occasionally been used to model compositional data in recent years [18]. State space models are useful for modeling a dynamic process that is observed only after the introduction of some measurement [e.g., 19]. Finally, we use the Gaussian process in our state space framework to help contend with irregularity in the sampling of our data. Rather than evolving in discrete jumps from one time point to the next, it allowed us to model the change in microbial logratio abundances as smoothly flowing through interruptions in observation. Other authors have made essentially the same choice, as in Äijö et al.’s TGP-CODA model [20]. In all, we suspect the results in this paper are robust to a *variety* of modeling decisions. A simple centered log-ratio ARIMA model, for example, yielded very similar estimates of ASV-ASV correlations (Figure 4 —figure supplement 1C).

Reviewer #3 (Recommendations for the authors):

• Good abstract, presentation, and introduction.

• Figure 2: perhaps mark in panel A what the threshold for significant positive/negative correlations was.

Because there is no threshold above which the correlations are all statistically significant, there is no easy way to represent significance on the heat map in Figure 2A. However, we have added a new supplementary figure (Figure 2 —figure supplement 2) that shows the same data as the heat map in Figure 2A, but with non-significant (FDR < 0.05) correlations blacked out.

• Positive correlation – as you note in several places – can be explained in (at least) two ways: the species have positive relations in some way (e.g. one is providing something necessary for the other), or the two simply like to be in the same kind of habitat, so when it is good for one it's also good for the other. You are aware of this, as both possibilities are mentioned in several places, but it seems that sometimes you choose to offer one and sometimes the other, with no clear reason (e.g. you propose that correlations at the phylum level are due to environmental preferences – lines 217-219 – but this explanation is in contrast to the strong emphasis on microbe-microbe interactions that is found throughout).

We have revised the text in several places to make sure we provide both explanations where relevant (see lines 118, 230, 351, etc.). For instance, the text you mention, which was originally at line 217-219, and now at lines 230 states, “This bias towards negative relationships is consistent with the expectation that neutral or negative relationships between ASVs are more common than mutualisms [21-23] and that more distantly related taxa (e.g., phyla) respond to distinct environmental drivers due to differences in metabolic requirements and lifestyles”. We also have added a much more detailed discussion of how we think these patterns contribute to our data starting at line 552.

• I would suggest having a discussion of these alternative explanations (and perhaps others) early on, and reference to this discussion in later interpretations of findings, throughout the results and Discussion sections.

(you are clearly aware of this, e.g. in line 407; I suggest discussing this topic in the introduction and referring to it throughout. This would help readers who aren't aware of the extensive research/discussion/debate about these questions in microbial ecology, landscape ecology, and elsewhere).

We added more thorough discussion of these alternatives in the Introduction (line 118 and line 230) and the Discussion (lines 552 to 567).

• A brief mention/clarification (at least) of causality vs. correlation would be a good idea in this context. Even if clear correlations are found between taxa, this doesn't imply causation, of course. Perhaps discuss in future directions the importance of intervention/manipulation studies to test for causation.

We added this idea to line 117, which now states, “…correlation cannot be used to infer causality, and in the absence of experiments, we cannot differentiate whether microbial correlation patterns arise from ecological interactions (e.g., competition, predation, facilitation) or shared responses to the environment.” Line 430 now also reads, “Without experiments, we cannot disentangle whether our observed bacterial correlations are due to ecological interactions between bacterial species or to shared responses to environmental gradients, either inside or outside the host.”

• There's quite a large literature in ecology, particularly microbial ecology, that deals with the link between pairwise interactions between bacteria within a larger consortium of species, and whether inferences can be made from pairwise interactions to more complex scenarios; consider referring to some of this literature and perhaps offering a discussion of your results in light of the insights proposed there. Some such studies (I'm not from the field, there may be better ones) are:

https://www.nature.com/articles/nature22898

https://onlinelibrary.wiley.com/doi/full/10.1111/ele.13211

https://www.nature.com/articles/s41559-017-0109

Also, have a look at one or two possibly relevant studies by Andrew Letten.

Thank you for pointing out the literature about this topic, and we agree with the value of citing it. We now cite several of these studies and briefly discuss the importance of future work that connects pairwise bacterial interactions to the emergent properties of the microbial community (see line 628).

• A possible interpretation of the finding that correlations, when exist, tend to be positive: if the driver of significant correlations is the environment, and not positive species' interactions, then this observation might be expected: pairs of species that share environmental preferences will be positively correlated, and pairs of species that prefer different environments would be uncorrelated (and not negatively correlated).

In other words: there is only one way in which environmental preferences can be similar, but many ways in which two environmental preferences can differ (and also an environment is similar to itself in all dimensions, but there are many dimensions in which two environments can differ). "All happy families are alike, but every unhappy family is unhappy in its own way (Leo Tolstoy, Anna Karenina, 1878)".

In a sense, this observation should thus perhaps be viewed as support of the hypothesis that the driver of the positive correlations you find is shared environmental preferences and not species-species interactions. I think. Consider.

Thank you for this interesting set of ideas! As mentioned above, we have added new emphasis to the idea that environmental preferences could be playing an important role in the patterns we observe, as an alternative to species-species interactions (see new discussion starting in line 351)—while also cautioning that our data set cannot unambiguously differentiate these possibilities (e.g., text starting at line 430). However, given our revised filtering criteria, we also no longer find a bias towards positive correlations in the most universal taxa (we still find that, overall, most correlations are negative).

• 545-555: If true, the positive correlations are due to shared preferences of environment, it perhaps makes sense that the children dataset, in which children differ quite a bit (more than pairs of baboons), shows a strong signal: the fact that children are different should create high diversity in the overall dataset, and when two children happen to be similar in the conditions they create in their guts – this (and the respective positive correlations between pairs of species that like these specific conditions) would stand out particularly significantly above all this noise. Maybe. This requires some deeper thought, so consider. ((this may be analogous to assessing heritability of traits – heritability seems to decrease – sometimes to the point of being non-significant/below detection level – in a homogenic population, and heritability estimates are higher when the population is diverse))

After correcting for the zero inflation in our data, we no longer see the bias towards positive relationships in the most universal correlation patterns. For this reason, we have not included the suggested ideas, but we agree that if correlated relationships are caused by environmental gradients, and if infants experience more environmental differences than baboons, it might be easier to detect significant correlations in an infant data set. However, the correlation patterns do not seem substantially different between baboons and infants, so we have chosen not to go there.

• 572 – 576 (starting with "We surmise that most") – I would be more cautious about this statement.

I tend to think that the driver of the correlation universality in your data is shared environmental preferences, and – apart from the point I made above – I think this is also particularly likely in light of the phylogenetic signal that you found (it makes sense that phylogenetically related species have similar environmental preferences, stemming from homology; this seems to me more parsimonious compared to the possibility that related species tend to be more supportive of one another for some reason, even though I can come up with some handwaving explanations that could support this if I really had to).

The "environment" in question is the one in the gut. Thus, controlling for diet or seasonal drivers is good, but far from ruling out that there are shared environments that are driving the signal; for that, you'd need to control for the extent to which pairs of host individuals tended to have more similar pH, hormonal status, immune activation (and its profile) and so on.

We agree. These ideas are discussed in other responses above, but briefly, we now state in the discussion, starting at line 559, that “our approach did not account for important environmental gradients within the gut, such as host immune profiles and intestinal pH. These factors also shape microbiome composition [e.g., 24, 25, 26], and can lead to shared abundance correlations between hosts even if hosts themselves differ. Ecological selection via within-host environments may explain our finding that genetic relatives share somewhat similar bacterial correlation patterns. Ecological selection is also consistent with our observation that the most consistent ASV-level correlations are between phylogenetically related taxa, and these patterns were strongest for positively associated taxon pairs. In support, phylogenetically related species have been shown to have similar environmental preferences [27].”

• 589: There seems to be a problem with this sentence. Look at the "the fact that…" – seems like something is missing.

This sentence has been revised to read, “Following recommended statistical practices [28], samples were not rarefied, but counts were agglomerated and transformed to additive log-ratios (ALR). Variation in sampling depth and relative abundance were modeled by the method described in a subsequent section.”

• Methods: I'd elaborate a bit further about the sequencing, e.g. whether you rarefied samples or accounted for uneven read counts in another way, and which 16s regions were amplified (and/or what their length was – amplifying just V3, for example, would lead to a very different ASV resolution from amplifying V3+V4).

We have expanded these sections of the methods. In line 648, we state “Following recommended statistical practices [28], samples were not rarefied, but counts were agglomerated and transformed to additive log-ratios (ALR). Variation in sampling depth and relative abundance were modeled by the method described in a subsequent section.”

To clarify our sequencing approach, we state in line 638 that, “The microbiome compositional profiles are derived from PCR amplification of a ~390 bp-long fragment that encompassed the V4 region of the 16S rRNA gene using primers 515F – 806R [29].”

References

- Silverman JD, Roche K, Holmes ZC, David LA, Mukherjee S. Bayesian Multinomial Logistic Normal Models through Marginally Latent Matrix-T Processes. Journal of Machine Learning Research. 2022;23:1-42.
- Quinn TP, Richarrson MF, Lovell D, Crowley TM. propr: An R-package for Identifying Proportionally Abundant Features Using Compositional Data Analysis Scientific Reports. 2017;7:16252.
- Cao Y, Lin W, Li H. Large covariance estimation for compositional data via compositionadjusted thresholding.. J Am Stat Assoc. 2019:759-72.
- Friedman J, Alm EJ. Inferring correlation networks from genomic survey data. PLoS Comput Biol. 2012;8(9):e1002687. doi: 10.1371/journal.pcbi.1002687. PubMed PMID: 23028285; PubMed Central PMCID: PMCPMC3447976.
- Risely A, Schmid DW, Muller-Klein N, Wilhelm K, Clutton-Brock TH, Manser MB, et al. Gut microbiota individuality is contingent on temporal scale and age in wild meerkats. Proc Biol Sci. 2022;289(1981):20220609. Epub 20220817. doi: 10.1098/rspb.2022.0609. PubMed PMID: 35975437; PubMed Central PMCID: PMCPMC9382201.
- Wilmanski T, Diener C, Rappaport N, Patwardhan S, Wiedrick J, Lapidus J, et al. Gut microbiome pattern reflects healthy ageing and predicts survival in humans. Nat Metab. 2021;3(2):274-86. Epub 20210218. doi: 10.1038/s42255-021-00348-0. PubMed PMID: 33619379; PubMed Central PMCID: PMCPMC8169080.
- Johnson AJ, Vangay P, Al-Ghalith GA, Hillmann BM, Ward TL, Shields-Cutler RR, et al. Daily Sampling Reveals Personalized Diet-Microbiome Associations in Humans. Cell Host and Microbe. 2019;25(6):789-802. Epub 2019/06/14. doi: 10.1016/j.chom.2019.05.005. PubMed PMID: 31194939.
- Franzosa EA, Huang K, Meadow JF, Gevers D, Lemon KP, Bohannan BJM, et al.Identifying personal microbiomes using metagenomic codes. Proceedings of the National Academy of Sciences. 2015;112(22):E2930-E8. doi: 10.1073/pnas.1423854112. PubMed PMID: WOS:000355832200014.
- Faith JJ, Guruge JL, Charbonneau M, Subramanian S, Seedorf H, Goodman AL, et al. The long-term stability of the human gut microbiota. Science. 2013;341(6141):1237439. Epub 2013/07/06. doi: 10.1126/science.1237439. PubMed PMID: 23828941; PubMed Central PMCID: PMC3791589.
- Bik EM, Costello EK, Switzer AD, Callahan BJ, Holmes SP, Wells RS, et al. Marine mammals harbor unique microbiotas shaped by and yet distinct from the sea. Nat Commun. 2016;7:10516. Epub 20160203. doi: 10.1038/ncomms10516. PubMed PMID: 26839246; PubMed Central PMCID: PMCPMC4742810.
- Caporaso JG, Lauber CL, Costello EK, Berg-Lyons D, Gonzalez A, Stombaugh J, et al. Moving pictures of the human microbiome. Genome Biology. 2011;12(5):R50. doi: Artn R50 Doi 10.1186/Gb-2011-12-5-R50. PubMed PMID: ISI:000295732700014.
- Costello EK, Lauber CL, Hamady M, Fierer N, Gordon JI, Knight R. Bacterial community variation in human body habitats across space and time. Science. 2009;326(5960):1694-7. doi: Doi 10.1126/Science.1177486. PubMed PMID: ISI:000272839000053.
- Dolinsek J, Goldschmidt F, Johnson DR. Synthetic microbial ecology and the dynamic interplay between microbial genotypes. Fems Microbiology Reviews. 2016;40(6):961-79. doi: 10.1093/femsre/fuw024. PubMed PMID: WOS:000387995000010.
- Louca S, Polz MF, Mazel F, Albright MBN, Huber JA, O'Connor MI, et al. Function and functional redundancy in microbial systems. Nat Ecol Evol. 2018;2(6):936-43. Epub 2018/04/18. doi: 10.1038/s41559-018-0519-1. PubMed PMID: 29662222.
- Rainey PB, Quistad SD. Toward a dynamical understanding of microbial communities. Philos Trans R Soc Lond B Biol Sci. 2020;375(1798):20190248. Epub 2020/03/24. doi: 10.1098/rstb.2019.0248. PubMed PMID: 32200735; PubMed Central PMCID: PMCPMC7133524.
- Martiny JB, Jones SE, Lennon JT, Martiny AC. Microbiomes in light of traits: A phylogenetic perspective. Science. 2015;350(6261):aac9323. doi: 10.1126/science.aac9323. PubMed PMID: 26542581.
- Debray R, Herbert RA, Jaffe AL, Crits-Christoph A, Power ME, Koskella B. Priority effects in microbiome assembly. Nat Rev Microbiol. 2022;20(2):109-21. Epub 20210827. doi: 10.1038/s41579-021-00604-w. PubMed PMID: 34453137.
- Gloor GB, Reid G. Compositional analysis: a valid approach to analyze microbiome highthroughput sequencing data. Can J Microbiol. 2016;62(8):692-703. Epub 2016/06/18. doi: 10.1139/cjm-2015-0821. PubMed PMID: 27314511.
- Joseph TA, Pasarkar AP, Pe'er I. Efficient and Accurate Inference of Mixed Microbial Population Trajectories from Longitudinal Count Data. Cell Syst. 2020;10(6):463-9 e6. Epub 20200624. doi: 10.1016/j.cels.2020.05.006. PubMed PMID: 32684275.
- Aijo T, Muller CL, Bonneau R. Temporal probabilistic modeling of bacterial compositions derived from 16S rRNA sequencing. Bioinformatics. 2018;34(3):372-80. doi: 10.1093/bioinformatics/btx549. PubMed PMID: 28968799; PubMed Central PMCID: PMCPMC5860357.
- Coyte KZ, Rao C, Rakoff-Nahoum S, Foster KR. Ecological rules for the assembly of microbiome communities. PLoS Biol. 2021;19(2):e3001116. Epub 20210219. doi: 10.1371/journal.pbio.3001116. PubMed PMID: 33606675; PubMed Central PMCID: PMCPMC7946185.
- Coyte KZ, Schluter J, Foster KR. The ecology of the microbiome: Networks, competition, and stability. Science. 2015;350(6261):663-6. doi: 10.1126/science.aad2602. PubMed PMID: 26542567.
- Palmer JD, Foster KR. Bacterial species rarely work together. Science. 2022;376(6593):581-2. Epub 20220505. doi: 10.1126/science.abn5093. PubMed PMID:35511986.
- Reese AT, Pereira FC, Schintlmeister A, Berry D, Wagner M, Hale LP, et al. Microbial nitrogen limitation in the mammalian large intestine. Nat Microbiol. 2018. Epub 2018/10/31. doi: 10.1038/s41564-018-0267-7. PubMed PMID: 30374168.
- Firrman J, Liu L, Mahalak K, Tanes C, Bittinger K, Tu V, et al. The impact of environmental pH on the gut microbiota community structure and short chain fatty acid production. FEMS Microbiol Ecol. 2022;98(5). doi: 10.1093/femsec/fiac038. PubMed PMID: 35383853.
- de Vos WM, Tilg H, Van Hul M, Cani PD. Gut microbiome and health: mechanistic insights. Gut. 2022;71(5):1020-32. Epub 20220201. doi: 10.1136/gutjnl-2021-326789. PubMed PMID: 35105664; PubMed Central PMCID: PMCPMC8995832.
- Tamames J, Sanchez PD, Nikel PI, Pedros-Alio C. Quantifying the Relative Importance of Phylogeny and Environmental Preferences As Drivers of Gene Content in Prokaryotic Microorganisms. Front Microbiol. 2016;7:433. Epub 20160331. doi: 10.3389/fmicb.2016.00433. PubMed PMID: 27065987; PubMed Central PMCID: PMCPMC4814473.
- Gloor GB, Macklaim JM, Pawlowsky-Glahn V, Egozcue JJ. Microbiome datasets are compositional: and this is not optional. Front Microbiol. 2017;8:2224. Epub 2017/12/01. doi: 10.3389/fmicb.2017.02224. PubMed PMID: 29187837; PubMed Central PMCID: PMCPMC5695134.
- Caporaso JG, Lauber CL, Walters WA, Berg-Lyons D, Lozupone CA, Turnbaugh PJ, et al. Global patterns of 16S rRNA diversity at a depth of millions of sequences per sample. Proceedings of the National Academy of Sciences. 2011;108:4516-22. doi: Doi 10.1073/Pnas.1000080107. PubMed PMID: ISI:000288451300002.
